# A double agent? Unveiling the chemical profile of the pathogenic fungus *Pyrrhoderma noxium* as an endophyte in true mangroves

**DOI:** 10.7717/peerj.20826

**Published:** 2026-02-20

**Authors:** Ming Han Han, Jing Sien Chang, Jen-Kit Tan, Swee Keong Yeap, Wei Lun Ng, Yoong Soon Yong

**Affiliations:** 1 China-ASEAN College of Marine Sciences, Xiamen University Malaysia, Sepang, Selangor, Malaysia; 2 Department of Biochemistry, Faculty of Medicine, Universiti Kebangsaan Malaysia, Cheras, Kuala Lumpur, Malaysia; 3 College of Ocean and Earth Sciences, Xiamen University, Xiamen, China; 4 R&D Quality Department, Osmosis Nutrition Sdn. Bhd., Nilai, Negeri Sembilan, Malaysia

**Keywords:** *Pyrrhoderma noxium*, Endophytic fungi, LC-MS/MS, Metabolites profile, Bioactive compounds

## Abstract

*Pyrrhoderma noxium*, commonly referred to as the brown root rot pathogen, has previously been recognized as a pathogenic species. However, it has been largely overlooked for its capability to produce useful bioactive compounds. In this study, we report for the first time that the fungus has been isolated as an endophytic fungus (EF) from the leaves of true mangrove plant species, and chemically profiled the fungal isolates to identify potential bioactive compounds. Three *P. noxium* isolates (*AA2AA*, *BG3BA* and *SA2AA*) were successfully identified *via* DNA barcoding and were subjected to methanolic extraction prior to chemical profiling via liquid chromatography-tandem mass spectrometry (LC-MS/MS). Despite proximity of the host plants, our results, comprising morphological characteristics, phylogenetic analysis, and the Jaccard Similarity Index based on the detected compounds showed that *AA2AA* and *SA2AA* possess greater similarity compared to any of them with *BG3BA*. Compounds produced by the *P. noxium* isolates were classified into six main classes (*i.e.*, amino acids and peptides, aromatics, terpenoids, phenolics, other lipids, and other alkaloids) and these compounds are believed to facilitate the equilibrium in endophyte-host interactions. According to literature, the identified compounds from the *P. noxium* isolates have previously been reported to possess anticancer, antioxidant, anti-inflammatory, antibacterial, antifungal, and antiviral activities, indicating significant potential for pharmaceutical applications. Besides, the chemical profiles of the *P. noxium* isolates determined in this study may serve as a reference for subsequent research on the biological control of *P. noxium* infection.

## Introduction

Mangroves are plants that thrive in the intertidal zones of tropical and subtropical regions (Otero et al., 2019), forming highly dynamic ecosystems that serve as significant biodiversity hotspots. The mangrove ecosystems support a diverse range of organisms, including endophytic fungi (EFs). These fungi reside within plant tissues such as leaves, stems, roots, and flowers (Muthukrishnan et al., 2022) without causing any pathogenic effects to their host plants (Baron & Rigobelo, 2022). Nevertheless, certain EFs exhibit pathogenicity in their natural environment. The existence of symbiotic relationships between EFs and their host plants is crucial for sustaining equilibria between these organisms. Given the challenging conditions of mangrove ecosystems such as high salinity, fluctuating oxygen levels, and intense microbial competition, EFs are believed to drive the synthesis of bioactive compounds as a survival strategy, similar to those synthesized by their host plants (Sopalun et al., 2021). The mangrove EFs have attracted attention for research due to their diversity, unique ecological characteristics, and most significantly, their capacity to synthesize novel bioactive compounds (Cadamuro et al., 2021).

*Pyrrhoderma noxium* (Corner) L. W. Zhou and Y. C. Dai, commonly referred to as the brown root rot pathogen, is recognized as a pathogenic fungus that has been reported to infect over 200 plant species, primarily distributed in tropical and subtropical regions (Panchalingam et al., 2022b). It targets the roots and lowest trunks of trees, causing extensive rot that typically results in tree mortality within a few months (Cannon et al., 2022). This fungal pathogen has been linked to significant economic losses in the agricultural and forestry sectors due to its aggressive pathogenicity, making it a major concern for plant health management (Adra et al., 2024). For instance, brown root rot disease was reported to cause the loss of 1,075 cashew trees in Indonesia, leading to a substantial yield loss of 5,106 kg of kernel, worth Rp20.5 million (Supriadi et al., 2004). In Australia, *P. noxium* was found to infect avocado trees, causing about 10% of trees to die in several infected orchards and leading to an estimated economic loss of $AUD5400 per hectare (Dann et al., 2009; Everett & Siebert, 2018). Numerous studies have explored biological control strategies against *P. noxium*, such as the antagonistic mechanisms of *Trichoderma* strains by Panchalingam et al. (2022b), and the application of metabolites produced by *Bacillus subtilis* by Liang et al. (2023). In addition to its pathogenic nature, *P. noxium* has the potential to produce a variety of bioactive compounds, which are secondary metabolites synthesized by fungi that exhibit a wide range of biological activities. For example, a study by Wang et al. (2023) extracted two terpenoids (3*β*,6*β*-dihydroxycinnamolide and 3*β*-hydroxycinnamolide) with anti-inflammatory properties from marine-derived *P. noxium*. This finding suggests that *P. noxium* may possess a broader repertoire of bioactive compounds with pharmaceutical relevance. However, limited research has explored the potential of endophytic *P. noxium* as a producer of useful bioactive compounds.

To identify the compounds produced by fungal cultures, a liquid chromatography-tandem mass spectrometry (LC-MS/MS) system is highly effective due to its high sensitivity and specificity (Wu & French, 2013). Hyphenated mass spectrometry analysis encompasses multiple stages of mass spectrometric evaluation, including the fragmentation of precursor ions *via* collision-induced dissociation (CID) and the measurement of the mass-to-charge ratio (*m/z*) of product ions (Sargent, 2013). The multiple monitoring mode (MRM) demonstrates high selectivity by reducing co-eluting interference from background noise (Shi et al., 2012). Prior research has shown that compounds belonging to classes such as alkaloids (Cui et al., 2017), polyketides (Chen et al., 2019), terpenoids (Zhang et al., 2017), steroids (Li et al., 2022), and lactones (Li et al., 2022) can be detected from the extracts of mangrove EFs. These bioactive compounds possess significant potential in drug discovery due to their anticancer, antioxidant, anti-inflammatory, antibacterial, antifungal, and antiviral activities (Cadamuro et al., 2021).

In this study, we report for the first time, the discovery of *P. noxium* as an endophytic fungus (EF) in true mangrove species. Being well-known for its pathogenic nature, the isolation of *P. noxium* from the leaves of true mangroves suggests a unique and previously unrecognized symbiotic relationship. The mechanisms underlying this association remain poorly understood, particularly on how *P. noxium* coexists with mangrove hosts without inducing visible disease symptoms. Given the limited knowledge on the chemical profile of endophytic *P. noxium*, this study aims to address the gap in understanding its potential bioactive compounds and their potential roles in mediating symbiotic interactions with its host. The findings of this study are expected to contribute to future research on natural product discovery and provide insights into the dual lifestyle and potential in biological control of pathogenic *P. noxium*.

## Materials and Methods

### Sample collection

Sampling was conducted during low tide, when the sandy mud regions were accessible, facilitating access to the mangrove foliage. The identification of mangrove tree species was carried out according to the “Mangrove Guidebook for Malaysia” published by Lee, Muhamad & Tong (2015) prior to leaf samplings. Three individuals were sampled from each mangrove species, including *Avicennia alba* Blume, *Sonneratia alba* J.E. Smith, *Rhizophora apiculata* Blume, *Bruguiera gymnorhiza* (L.) Lamk., and *Scyphiphora hydrophyllacea* Gaertn. f., at Port Dickson, Negeri Sembilan, Malaysia (2.4925499°N, 101.8381413°E). From each individual, a branch with at least two leaves still attached, was sampled. Using visual inspection, the leaves were made sure to be devoid of damage and infection. The samples were kept in individual zip-lock bags, transported to the laboratory in a cool box, and processed within 24 h. Plant voucher specimens were deposited in the Mycology Laboratory, China-ASEAN College of Marine Sciences, Xiamen University Malaysia, under the following numbers: *A. alba* Blume (CAMS-MRG-001), *S. alba* J.E. Smith (CAMS-MRG-002), *R. apiculata* Blume (CAMS-MRG-003), *B. gymnorhiza* (L.) Lamk. (CAMS-MRG-004), and *S. hydrophyllacea* Gaertn. f. (CAMS-MRG-005).

### Isolation and cultivation of mangrove EFs

At the laboratory, two leaves were harvested from each sampled branch for surface sterilization to eliminate any debris and microorganisms that may be present on the leaf surfaces. The leaves were initially rinsed under running tap water and subsequently dried with tissue paper. A small segment of each leaf, encompassing the midrib, was excised with a surgical blade. Using the protocol by Hamzah et al. (2018) with minor modifications to the solvent concentrations, immersion durations, and the addition of an extra immersion step, the leaf segments were surface-sterilized by sequential immersion in 75% ethanol for 1 min, 1.8% sodium hypochlorite solution (∼35% Clorox bleach) for 20 min, and sterilized ultrapure water (ddH_2_O) for 5 min. Subsequently, the leaves were taken out and rinsed again with sterilized ddH_2_O. The peripheries of the sterilized leaves were then trimmed with a surgical blade. Five leaf fragments measuring 0.5 cm × 0.5 cm were cut out from each trimmed leaf segment. The five leaf fragments comprised the four corners of the leaf and fragment with the midrib. The leaf fragments were incubated on a potato dextrose agar (PDA) supplemented with 100 mg/L of chloramphenicol for a period of two weeks. In this period, fungi that grow out of the edges of the leaf fragments were considered as EFs. The EFs acquired from the leaves were initially streaked onto a fresh PDA plate to acquire pure isolates. Subsequently, single-colony isolation was performed by transferring an individual colony of EF from the streaked plate to a fresh PDA plate. Colony characteristics were described based on Kołwzan, Adamiak & Rybak (2011), and the aspects that were described included front and reverse colors, textures, margins, forms, and elevations. Photographs of each EF isolate growing on PDA (front and reverse) were captured on the seventh day for documentation purposes.

### DNA extraction, PCR and sequencing

Total DNA of each EF isolate was first extracted using a modified EtNa gDNA extraction method by Vingataramin & Frost (2015). First, an EtNa solution was prepared by mixing 5.5 mL of 2 M sodium hydroxide (NaOH), five mL of 0.025 M ethylenediaminetetraacetic acid (EDTA), and 35 mL of 96% EtOH together. A small amount of EF was scraped using a sterile pipette tip and then transferred into a 1.5 mL centrifuge tube containing 50 µL of sterile ddH_2_O. Next, 230 µL of EtNa solution was added and mixed, then incubated at 80 °C for 10 min. After that, centrifugation was carried out at 13,700 × g for 10 min, and the supernatant was discarded. 30 µL of Tris-EDTA (TE) buffer was added to the DNA pellet and resuspended to obtain the crude DNA stock. The crude DNA was kept at −20 °C until use.

PCR amplification was carried out to amplify the internal transcribed spacer (ITS) region of the mangrove EFs using the primers ITS1F (5′-CTTGGTCATTTAGAGGAAGTAA-3′) and ITS4 (5′-TCCTCCGCTTATTGATATGC-3′) (Raja et al., 2017). A total final reaction volume of 25 µL mixture was prepared, consisting of one µL of DNA, one µM of each primer and 1 × NEXpro™ e PCR 2X Master Mix (NEX Diagnostics, Korea). The PCR programme consisted of an initial denaturation for 10 min at 95 °C; 30 cycles of denaturation for 30 s at 95 °C, annealing for 30 s at 50 °C, and extension for 1 min at 72 °C; and a final extension for 5 min at 72 °C. Agarose gel electrophoresis was carried out to check for PCR amplification success, and PCR amplicons were sequenced on an ABI3730 platform (Applied Biosystems, MA, US). The resultant DNA sequences were matched against records in the National Center for Biotechnology Information (NCBI) GenBank database using the Basic Local Alignment Search Tool (BLAST: https://blast.ncbi.nlm.nih.gov/Blast.cgi; accessed on 2 May 2023). The identity of each EF isolate in this study was assigned based on the criteria that the percent identities must be >99%. Isolates identified as *P. noxium* were specifically chosen for the subsequent analyses.

### Phylogenetic analysis

Phylogenetic analysis was conducted through the Molecular Evolutionary Genetics Analysis (MEGA) version 11 software (Tamura, Stecher & Kumar, 2021) to understand the genetic relationships of the *P. noxium* isolated in this study. Initially, multiple DNA sequences of *P. noxium* were retrieved from the NCBI GenBank database (GenBank accession numbers: MG645130.1, LC743760.1, HQ400701.1, MZ853976.1, LN558874.1, PP504518.1, MW543018.1, MT804574.1, KU194338.1, OP785060.1, MK440618.1, OQ076504.1, KM079594.1 and OR888991.1). An outgroup species, *Spiromyces aspiralis* (GenBank accession number: NR_119554.1), was included in the analysis. Alignment of the DNA sequences were conducted using ClustalW (Thompson, Higgins & Gibson, 1994), and a phylogenetic tree was constructed using the Neighbor-Joining (NJ) method (Saitou & Nei, 1987). The evolutionary distances were computed using the Maximum Composite Likelihood method (Tamura, Nei & Kumar, 2004) with all positions with gaps and missing data excluded.

### Biochemical analysis using tandem mass spectrometry

Methanol containing 0.005% butylated hydroxytoluene (BHT) was employed for the extraction of bioactive compounds for LC-MS/MS analyses. Agars containing *P. noxium* were initially transferred into a 200 mL conical flask. Subsequently, 100 mL of methanol was added into the conical flask for overnight extraction. Following, the extract was collected, while the fungus was re-extracted with the fresh solvent for another two times. Eventually, extracts from the same sample were pooled and dried with nitrogen flow. Dried extracts were kept at −80 °C.

Before the LC-MS/MS analysis, *P. noxium* extracts were reconstituted in 500 µL of methanol and filtered using a 0.22 µm PTFE syringe filter. Extracts were analyzed utilising a reversed phase Kinetex F5 column (1.7 µm, 2.1 mm ×100 mm; Phenomenex, Torrance, CA, USA) for chromatographic separation, as detailed in Yong et al. (2017), with minor modifications. Briefly, five µL of extract was injected into a Dionex UltiMate 3000 Quaternary Ultra-High-Performance Liquid Chromatography (UHPLC) system (Thermo Fisher Scientific, Waltham, MA, USA) connected to a Thermo Scientific Q Exactive HF Orbitrap mass spectrometry system (Thermo Fisher Scientific, Waltham, MA, USA), which featured a heated electrospray ionization (HESI) probe. The reversed phase column was maintained at 35 °C with a flow rate of 450 µL/min throughout the analysis. The mobile phases consisted of solvent A (water with 0.1% formic acid and 1% of 10 mM ammonium formate) and solvent B (a mixture of acetonitrile and methanol in a 6:4 v/v ratio, containing 0.1% of formic acid and 1% of 10 mM ammonium formate). The gradient elution was linearly increased from 1% to 70% solvent B in 7 min, followed by 100% solvent B in 10 min and maintained for 3 min. The column was subsequently conditioned as initial for one minute prior to the subsequent injection. Data dependent acquisition (DDA) data were acquired in positive and negative modes as described in Teoh et al. (2023), and the instrument was calibrated using Pierce LTQ ESI Positive Calibrations solution and Pierce LTQ ESI Negative Calibrations solution (Thermo Fisher Scientific, Waltham, MA, USA) before analysis. The acquired data were pre-processed using the Thermo Scientific Compound Discoverer 3.3 software (Thermo Fisher Scientific, Waltham, MA, USA) with the default workflow for natural products analysis (Ng et al., 2024b). Following, the identification of compounds was conducted by matching the acquired MS/MS data of each feature against the mzCloud database. Unmatched features were reprocessed using the ChemSpider database (Pence & Williams, 2010) and filtered with a FISh score above 50. The confidence level for compound identification was assigned based on the criteria proposed by Summer et al. (2007), whereby compounds matched against reference MS/MS spectra in the mzCloud database were classified as Level 2 (probable structure) identifications, while those annotated through Chemspider and filtered by FISh score >50 were categorized as Level 3 (tentative candidates).

### Similarity index based on compounds produced

Upon identification of the bioactive compounds in the *P. noxium* samples, they were initially categorized based on their compound classes. A similarity test was conducted utilizing the Jaccard Similarity Index, comparing *P. noxium* with compounds generated through Paleontological Statistics (PAST) software version 4.15, based on the presence or absence of detected compounds. This preliminary similarity assessment serves to quantify the chemical similarity among the three different *P. noxium* isolates based on their identified compounds.

### Literature search on potential bioactivities of compounds

The potential bioactivities of compounds extracted from *P. noxium*, including anticancer, antioxidant, anti-inflammatory, antibacterial, antifungal, and antiviral properties, were assessed by reviewing published journal articles *via* Google Scholar and PubMed. The keywords employed to investigate the bioactivities of the compounds are “Name of compound + anticancer/antioxidant/anti-inflammation/antibacterial/antifungal/antiviral”. The literature search aimed to highlight the pharmacological potential of *P. noxium*, providing valuable insights into its bioactive compounds.

## Results and Discussions

### Identification of *P. noxium* Isolates

**Figure 1:**
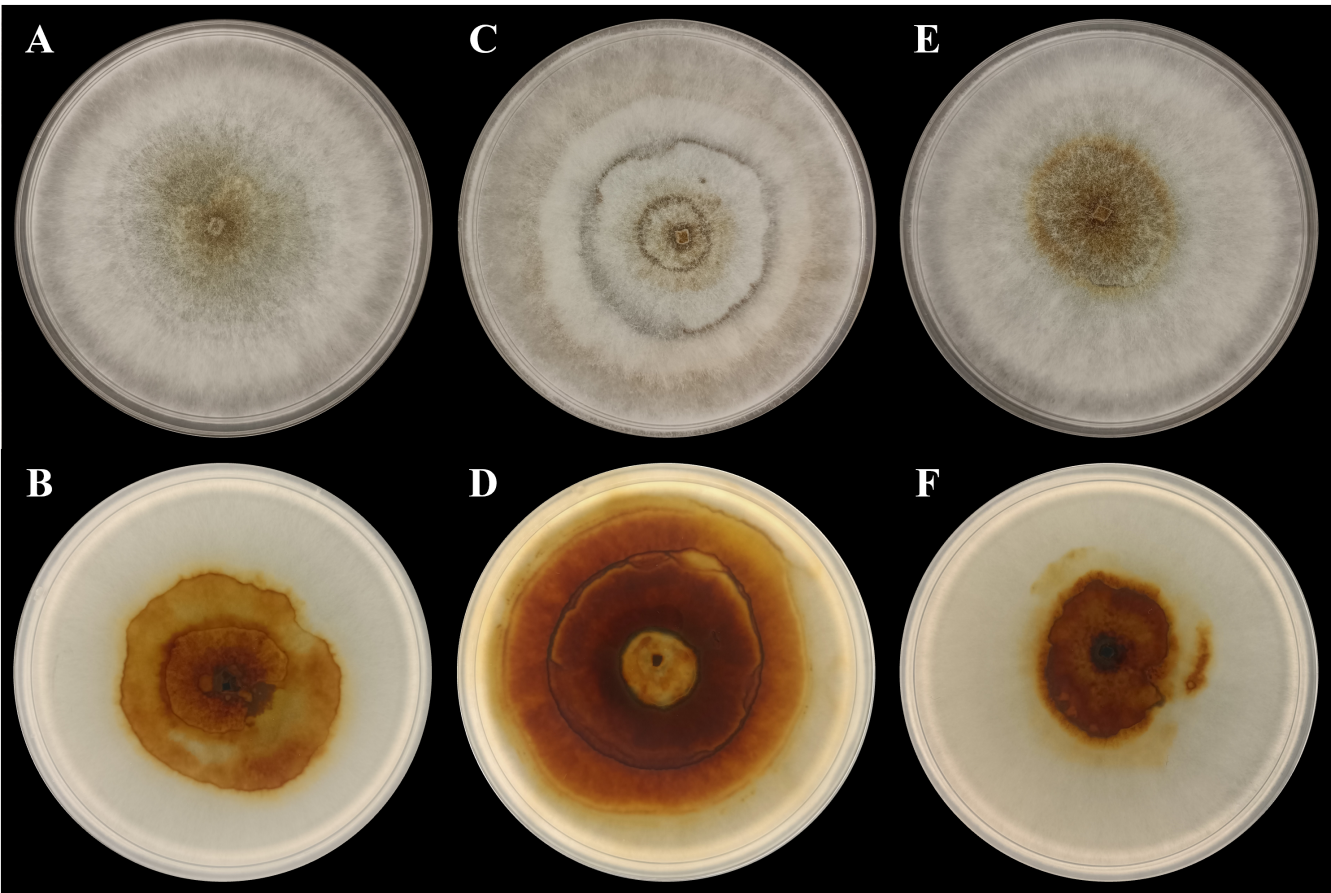
Photographs of plate cultures for the three *P. noxium* captured on Day-7. (A) *AA2AA* (front), (B) *AA2AA* (reverse), (C) *BG3BA* (front), (D) *BG3BA* (reverse), (E) *SA2AA* (front) and (F) *SA2AA* (reverse).

**Figure 2:**
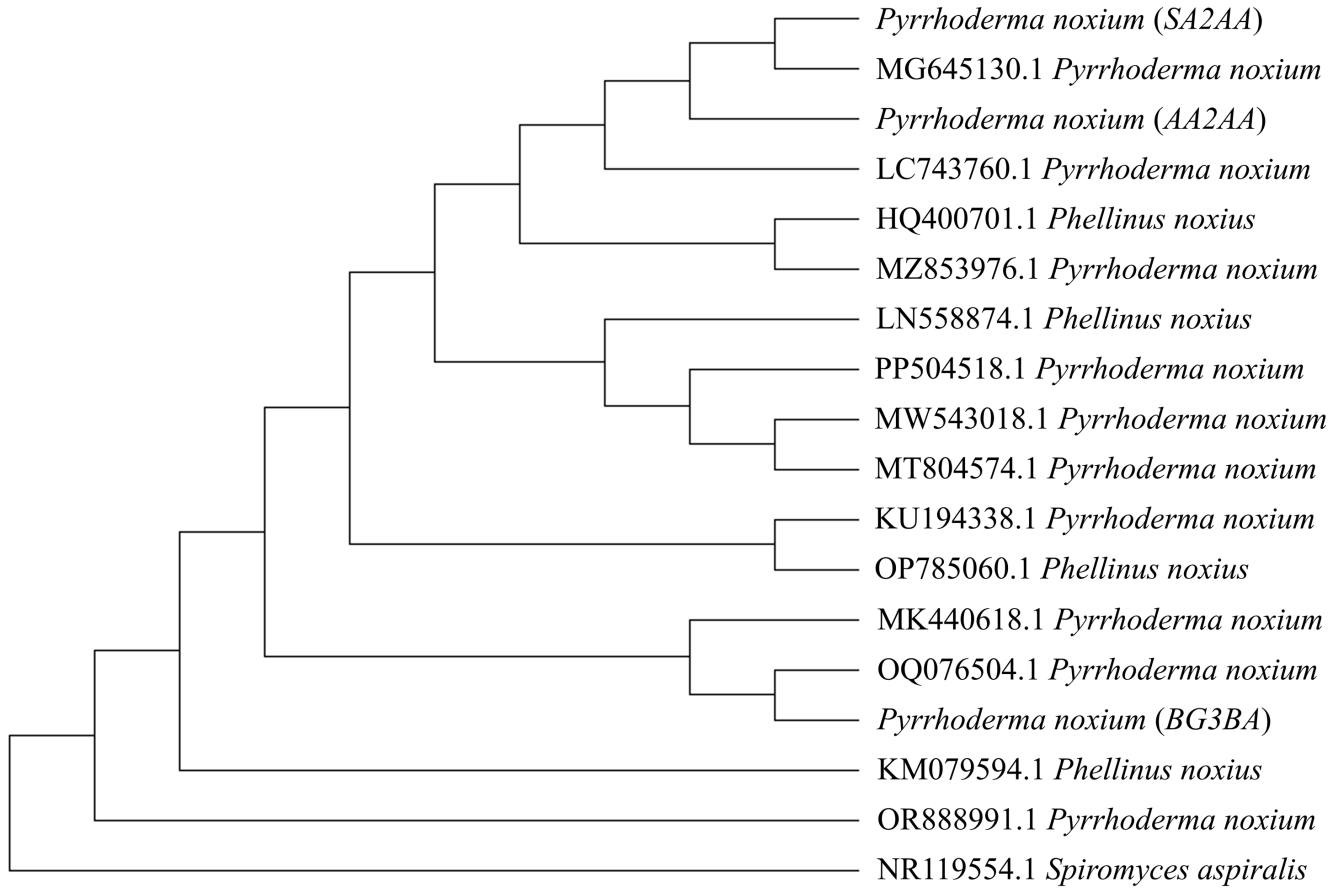
Phylogenetic tree constructed using the Neighbor-Joining (NJ) method. A total of 14 previously reported *P. noxium* (or *Phellinus noxius*) accessions along with three *P. noxium* accessions obtained in this study were shown. *Spiromyces aspiralis* is included as an outgroup to root the phylogeny.

In the present study, three *P. noxium* isolates were isolated from *A. alba* (isolate *AA2AA*), *B. gymnorhiza* (isolate *BG3BA*), and *S. alba* (isolate *SA2AA*), respectively. The DNA sequences used to identify the isolates have been deposited in NCBI GenBank, with the accession numbers PQ821345, PQ821348, and PQ821349 (see Table 1 for the NCBI BLAST results). The results showed that *AA2AA* and *SA2AA* matched the same accession on NCBI GenBank, although they were isolated from different mangrove host plants. By viewing the 7-day-old front and reverse plate culture photographs of all three *P. noxium* samples (Fig. 1), *AA2AA* and *SA2AA* also exhibit similar colony morphological characteristics, whereas *BG2BA* differs noticeably. The surfaces of *AA2AA* and *SA2AA* colonies show a smooth transition from the center to the edge of the agar plates, while *BG3BA* features several distinct concentric circles as it expands outward. Furthermore, the reverse sides of *AA2AA* and *SA2AA* have smaller brown patches as compared to *BG2BA*, which has a larger brown patch with a lighter center. The detailed descriptions of colony morphological characteristics for the three *P. noxium* isolates were tabulated in Table 2. Apart from that, these observations were further supported by the Neighbor-Joining tree shown in Fig. 2 where *AA2AA* and *SA2AA* are genetically closer to each other compared to *BG3BA*. According to Voordeckers et al. (2012), fungal colony morphology is regulated by a complex genetic network that involves multiple signalling cascades such as MAPK, TORC, SNF1, and RIM101 pathways. These pathways control key aspects of fungal growth, differentiation, and environmental adaptation. As a result, variations in gene expression led to morphological differences, ultimately causing strain differentiation within the same fungal species.

**Table 1:** NCBI GenBank search results for the three *P. noxium* isolates obtained in this study.

*P. noxium* isolate	Query cover	Percent identity	No. of bp used	Highest match on GenBank (accession number)
*AA2AA*	95%	99.19%	643	HQ400701.1
*BG3BA*	100%	99.70%	657	KU194338.1
*SA2AA*	95%	99.19%	643	HQ400701.1

**Table 2:** Descriptions of colony morphological characteristics for the three *P. noxium* isolates.

*P. noxium* isolate	Colony morphological characteristics
*AA2AA*	• Surface colour: Light brown to white • Reverse colour: Brown to white • Texture: Smooth • Margin: Ciliate • Form: Round with radiating margin • Elevation: Flat
*BG3BA*	• Surface colour: Light brown to white • Reverse colour: Light brown (center) to brown to white • Texture: Smooth • Margin: Ciliate • Form: Round with radiating margin • Elevation: Flat
*SA2AA*	• Surface colour: Brown to white • Reverse colour: Brown to white • Texture: Smooth • Margin: Ciliate • Form: Round with radiating margin • Elevation: Flat

Hoffman & Arnold (2008) demonstrated that geographic locality may play a more important role than host identity in shaping the fungal endophyte communities, *i.e.,* different hosts from the same site usually share similar endophytes when compared to plant hosts of the same species located at different sites. This could be attributed to factors such as climate (Koide, Ricks & Davis, 2017) and insect activity (Devarajan & Suryanarayanan, 2006), which may facilitate the dispersal of EFs among nearby hosts. In this study, the sampled *A. alba* and *S. alba* occurred close to each other and were more seaward, while *B. gymnorhiza* was more landward. Therefore, dispersal of *P. noxium* may have occurred between *A. alba* and *S. alba*, and could have contributed to the higher similarity observed between their *P. noxium* isolates (*AA2AA* and *SA2AA*). However, additional analyses are required to understand the specific processes that may have shaped the observed genetic patterns in Fig. 2. The clustering of isolates *AA2AA* and *SA2AA* with an isolate from Australia (accession number: MG645130.1), and *BG3BA* with an isolate from Thailand (accession number: OQ076504.1) may suggest the possible influence of regional dispersal on *P. noxium* population structure. Nonetheless, this observation remains preliminary due to the limited number of sequences analyzed.

### Compound classes in endophyte-host relationships

**Figure 3:**
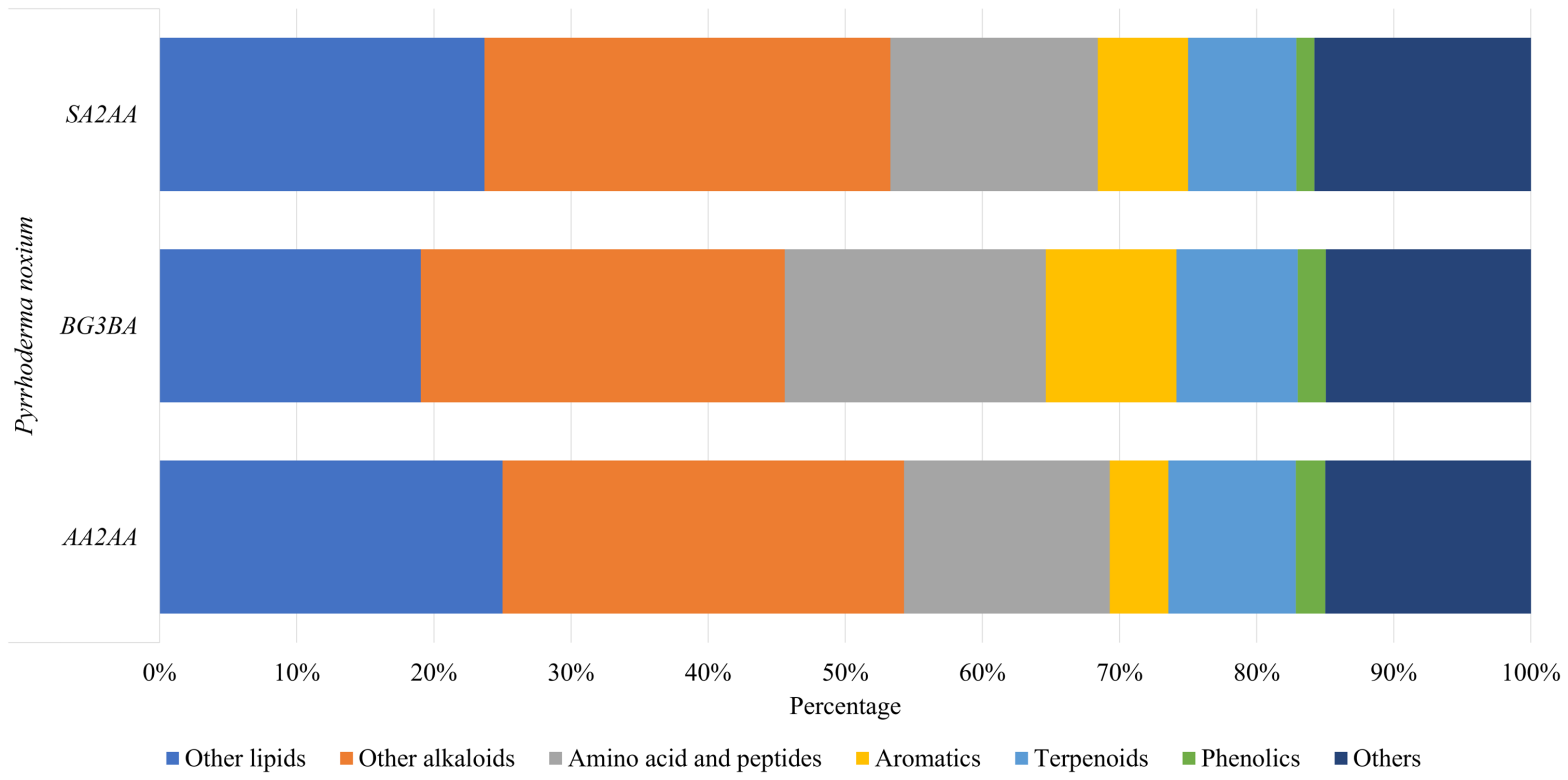
Bar chart displaying the percentage of main compound classes identified from three different *P. noxium* extracts, with each color represents a distinct compound class. The main compound classes identified are amino acids and peptides, aromatics, terpenoids, phenolics, other lipids, and other alkaloids.

A total of 199 compounds were identified (Supplemental Information 1—Compound list of *Pyrrhoderma noxium*) by LC-MS/MS analyses and were grouped into six main compound classes—the amino acids and peptides, aromatics, terpenoids, phenolics, other lipids, and other alkaloids. The number and percentage of compounds in each major compound class were tabulated (see Supplemental Information 2—Number and Percentage of Compounds in Each Major Compound Class), and the percentages of main compound classes identified from the *P. noxium* are shown in Fig. 3. In all three *P. noxium* samples, these six main compound classes accounted for about 85% or more of the total compounds identified. However, it should be noted that the LC-MS/MS analyses were conducted with a single replicate, as the study aimed to qualitatively screen for potential bioactive compounds rather than to perform quantitative or targeted compound identification.

Compounds produced by the EFs play an important role in endophyte-host symbiotic relationships. These compounds not only benefit the fungi themselves but can also benefit their host plants. For example, they can promote growth of plants (Poveda et al., 2021), increase the plant resistance to pathogens (Singh & Kumar, 2023), and improve resistance to abiotic stresses such as oxidative stress (Ogbe, Finnie & Van Staden, 2020). On the other hand, EFs use these compounds to compete, survive, and enhance colonization within the host plants; EFs have been shown to not only produce compounds that inhibit the growth of their competitors and neutralize the toxins produced by competitors, but also form symbiotic alliances with their hosts that allow EFs to survive in harsh environments (Alam et al., 2021).

### Similarity index based on compounds produced

The Jaccard coefficient, also known as Intersection over Union, is often used to measure the similarity between two objects (Survarachakan et al. (2022). From the results, *AA2AA* and *SA2AA* showed greater similarity to each other, with Jaccard Similarity Index of 0.62, as compared to their respective comparisons with *BG3BA* (Table 3). From our results, the similarity analysis based on the compounds produced agreed with the observations from colony morphological characteristics and phylogenetic tree analysis (Table 2 and Fig. 2). Previous studies have demonstrated the utility of chemotaxonomy *via* clustering of chemical profiles, *i.e.,* from plants (Lee, Yoo & Yang, 2021) and algae (Ng et al., 2024a), based on the fact that species tend to consistently produce species-specific profiles of metabolites (Frisvad, Andersen & Thrane, 2008).

**Table 3:** Jaccard similarity indices based on compounds produced by the three different *P. noxium* isolates.

	*AA2AA*	*BG3BA*	*SA2AA*
*AA2AA*	1.00		
*BG3BA*	0.51	1.00	
*SA2AA*	0.62	0.53	1.00

The greater similarity between the chemical profiles of *AA2AA* and *SA2AA* may be influenced by shared environmental factors, *i.e.,* salinity, tidal exposure, and substrate conditions (Alami et al., 2024; Ma et al., 2023). These shared environmental factors likely impose similar physiological stresses on their respective hosts, indirectly shaping the metabolic responses of their associated fungi. Moreover, *P. noxium* isolates with closer genetic relationships may possess a conserved core metabolomic profile, as fungal biosynthetic pathways tend to be evolutionarily conserved. This is supported by the findings by Bruna et al. (2024), who reported that closely related strains shared similar biosynthetic gene cluster types. Consequently, while genetic similarity provides a foundation for conserved metabolite production, environmental factors may therefore exert a stronger influence in modulating secondary metabolite expression. Together, these factors could explain the higher similarity in the chemical profiles of *AA2AA* and *SA2AA* compared to *BG3BA.*

### Potential bioactivities of bioactive compounds identified in *P. noxium*

Bioactive compounds produced by EFs have attracted significant attention due to their diverse pharmacological properties, including anticancer, anti-inflammatory, antioxidant, antibacterial, antifungal, and antiviral activities. These properties are particularly relevant for drug discovery and biomedical applications, as natural compounds derived from fungi have been the foundation of many clinically approved drugs. Forty-six bioactive compounds identified from the *P. noxium* extracts were found to have potential bioactivities according to literature (Supplemental Information 3—Summary Table for the Potential Bioactivities of Compounds Extracted from *Pyrrhoderma noxium*; with further details of the potential bioactivities summarized in Supplemental Information 4—Detailed Potential Bioactivities of Compounds Extracted from *Pyrrhoderma noxium*). In the present study, 18 bioactive compounds with anticancer potential were identified from *P. noxium*, which have been shown to have effects in various cancer cell lines (Supplemental Information 4—Table S1). As far as we are aware, our study is the first to document potential anticancer potential of *P. noxium* based on the compounds it produces.

Moreover, 20 compounds were found to possess antioxidant properties (Supplemental Information 4—Table S2). This is also the first time that *P. noxium* has been reported to potentially harbor antioxidant properties. A total of 24 compounds were identified to have anti-inflammatory potential in this study (Supplemental Information 4—Table S3). Bioactive compounds with anti-inflammatory activities, 3*β*,6*β*-dihydroxycinnamolide and 3*β*-hydroxycinnamolide, were previously identified by Wang et al. (2023) from marine sediment-derived *P. noxium*. Besides, a total of 12 compounds were found to exhibit antibacterial activities against various bacteria (Supplemental Information 4—Table S4). To date, no studies have been conducted on antibacterial compounds produced by *P. noxium*. A total of 10 compounds were identified with antifungal potential against various fungal species (Supplemental Information 4—Table S5). No previous studies have explored antifungal compounds produced by *P. noxium*. Only six compounds were identified to have antiviral potential against various viruses (Supplemental Information 4—Table S6). There is currently no published research on antiviral compounds produced by *P. noxium*.

### Potential implications for biological control of pathogenic *P. noxium*

In this study, the first discovery of *P. noxium* as an EF in true mangrove plant species broadens our understanding of its interactions with host plants. Often observed as a pathogenic fungus residing within host plants, *P. noxium* is considered an opportunistic pathogen (EFSA Panel on Plant Health et al., 2024). This means that *P. noxium* typically infects weakened or damaged plants while remaining harmless when colonizing healthy host tissues. Although primarily known for causing the brown root rot disease, the EFSA report notes that *P. noxium* has been isolated as an endophyte from a few plant species, indicating that it can persist in a symptomless or latent state within certain hosts. Recognizing this dual lifestyle, in which the fungus can exist both endophytically and as an opportunistic pathogen, highlights the potential role of compounds produced by *P. noxium* in modulating host defenses and maintaining a balanced host-fungus relationship. Compounds produced during its endophytic phase may help sustain equilibrium with the host, but disruptions in this balance can reduce host resistance, making the plant more susceptible to pathogens, including opportunistic pathogens such as *P. noxium*. Furthermore, the wide host range of this pathogen, combined with its ability to survive endophytically in asymptomatic plants, complicates early detection and limits the feasibility of developing standard diagnostic protocols for all potential host.

The chemical profiles of *P. noxium* generated *via* LC-MS/MS analyses generated in this study can serve as a reference for future researchers studying the biological control of *P. noxium* infection. Previous studies on the biological control of *P. noxium* infection focused on the use of different fungi and bacteria or their combination. The most commonly used fungal species was *Trichoderma* sp., while *Streptomyces* sp. was the most commonly used bacterial species. According to Panchalingam et al. (2022b), two *Trichoderma* strains demonstrated mycoparasitic ability against *P. noxium,* a form of antagonism involving direct physical contact with the *P. noxium* mycelium. On the other hand, Adra et al. (2024) showed that four *Streptomyces* isolates obtained from termite gut could be used as biological control agent through the production of antifungal volatile compounds, siderophores, and indole acetic acid. Furthermore, the consortium of *Trichoderma* and *Streptomyces* proved more effective in inhibiting *P. noxium* growth than individual biocontrol agents (Panchalingam et al., 2022a). Based on our findings (Supplemental Information 4—Tables S4 and S5), the chemical profiling of *P. noxium* did not identify bioactive compounds known to target *Trichoderma* sp. and *Streptomyces* sp., supporting the potential utility of these biological control agents against *P. noxium*.

## Conclusions

This study isolated endophytic *P. noxium* from three true mangrove plant species, namely *A. alba*, *B. gymnorhiza*, and *S. alba*. While sharing the same species identity based on molecular identification, the three *P. noxium* isolates exhibited distinct colony morphological characteristics and metabolite profiles, suggesting possible host-dependent metabolic variation. The LC-MS/MS screening revealed a broad spectrum of bioactive compounds, highlighting the capacity of *P. noxium* to synthesize a variety of compounds beyond its known pathogenic role. Collectively, these findings expand the ecological understanding of *P. noxium* as a mangrove endophyte and underscore its prospects in pharmaceutical and biological control applications. Future studies should aim to purify and characterize the detected metabolites and explore their specific biological activities. Additionally, comparative genomic and transcriptomic analyses could provide deeper insights into the biosynthetic pathways for metabolite production and host-specific adaptations in *P. noxium*.

## Supplemental Information

10.7717/peerj.20826/supp-1 Supplemental Information 1 Compound List of *Pyrrhoderma noxium* The compounds detected by LC-MS/MS in all three Pyrrhoderma noxium

10.7717/peerj.20826/supp-2 Supplemental Information 2 Number and Percentage of Compounds in each Major Compound Class number and percentage of compounds in each major compound class

10.7717/peerj.20826/supp-3 Supplemental Information 3 Summary Table for the Potential Bioactivities of Compounds Extracted from *Pyrrhoderma noxium* Summary tables for the potential bioactivities of compounds extracted from P. noxium

10.7717/peerj.20826/supp-4 Supplemental Information 4 Detailed Potential Bioactivities of Compounds Extracted from *Pyrrhoderma noxium* The literature search were done on the detected compounds on their potential bioactivities and the potential bioactivities

10.7717/peerj.20826/supp-5 Supplemental Information 5 Total Ion Chromatograms Acquired from LC-MS/MS The total ion chromatograms acquired from LC-MS/MS

## Additional Information and Declarations

### DNA Deposition

The following information was supplied regarding the deposition of DNA sequences: The raw sequence data are available at NCBI GenBank: PQ821345, PQ821348, PQ821349.

### Data Availability

The following information was supplied regarding data availability: The data is available in the Supplemental Files. The LC-MS/MS data is available at Zenodo: Han, M. H., Chang, J. S., Yeap, S. K., Ng, W. L., & Yong, Y. S. (2025). A double agent? Unveiling the chemical profile of the pathogenic fungus Pyrrhoderma noxium as an endophyte in true mangroves [Data set]. Zenodo. https://doi.org/10.5281/zenodo.17333866
The raw sequence data are available at NCBI GenBank: PQ821345, PQ821348, PQ821349.

## References

[ref-1] Adra C, Panchalingam H, Foster K, Tomlin R, Hayes RA, Kurtböke DI (2024). *In vitro* biological control of *Pyrrhoderma noxium* using volatile compounds produced by termite gut-associated streptomycetes. Frontiers in Plant Science.

[ref-2] Alam B, Liˇ J, Gě Q, Khan MA, Gōng J, Mehmood S, Yuán Y, Goˇng W (2021). Endophytic fungi: from symbiosis to secondary metabolite communications or vice versa?. Frontiers in Plant Science.

[ref-3] Alami MM, Guo S, Mei Z, Yang G, Wang X (2024). Environmental factors on secondary metabolism in medicinal plants: exploring accelerating factors. Medicinal Plant Biology.

[ref-4] Baron NC, Rigobelo EC (2022). Endophytic fungi: a tool for plant growth promotion and sustainable agriculture. Mycology.

[ref-5] Bruna P, Núñez-Montero K, Contreras MJ, Leal K, García M, Abanto M, Barrientos L (2024). Biosynthetic gene clusters with biotechnological applications in novel Antarctic isolates from *Actinomycetota*. Applied Microbiology and Biotechnology.

[ref-6] Cadamuro RD, da Silveira Bastos IMA, Silva IT, da Cruz ACC, Robl D, Sandjo LP, Alves Jr S, Lorenzo JM, Rodríguez-Lázaro D, Treichel H, Steindel M, Fongaro G (2021). Bioactive compounds from mangrove endophytic fungus and their uses for microorganism control. Journal of Fungi.

[ref-7] Cannon PG, Klopfenstein NB, Kim M-S, Stewart JE, Chung C-L (2022). Brown root rot disease caused by *Phellinus noxius* in US-affiliated Pacific Islands.

[ref-8] Chen Y, Yang W, Zou G, Chen S, Pang J, She Z (2019). Bioactive polyketides from the mangrove endophytic fungi *Phoma* sp. SYSU-SK-7. Fitoterapia.

[ref-9] Cui H, Yu J, Chen S, Ding M, Huang X, Yuan J, She Z (2017). Alkaloids from the mangrove endophytic fungus *Diaporthe phaseolorum* SKS019. Bioorganic & Medicinal Chemistry Letters.

[ref-10] Dann E, Smith L, Pegg K, Grose M, Pegg G (2009). Report on *Phellinus noxius*, the cause of brown rot, in Australian avocados. Talking avocados.

[ref-11] Devarajan P, Suryanarayanan T (2006). Evidence for the role of phytophagous insects in dispersal of non-grass fungal endophytes. Fungal Diversity.

[ref-12] Bragard C, Baptista P, Chatzivassiliou E, Di Serio F, Gonthier P, Jaques Miret JA, Justesen AF, MacLeod A, Magnusson CS, Milonas P, Navas-Cortes JA, Parnell S, Potting R, Stefani E, Thulke HH, Van der Werf W, Vicent Civera A, Yuen J, Zappalà L, Golic D, Gobbi A, Maiorano A, Pautasso M, Reignault PL, EFSA Panel on Plant Health (2024). Pest categorisation of *Pyrrhoderma noxium*. EFSA Journal.

[ref-13] Everett KR, Siebert B (2018). Exotic plant disease threats to the New Zealand avocado industry and climatic suitability: a review. New Zealand Plant Protection.

[ref-14] Frisvad JC, Andersen B, Thrane U (2008). The use of secondary metabolite profiling in chemotaxonomy of filamentous fungi. Mycological Research.

[ref-15] Hamzah TNT, Lee SY, Hidayat A, Terhem R, Faridah-Hanum I, Mohamed R (2018). Diversity and characterization of endophytic fungi isolated from the tropical mangrove species, *Rhizophora mucronata*, and identification of potential antagonists against the soil-borne fungus, *Fusarium solani*. Frontiers in Microbiology.

[ref-16] Hoffman MT, Arnold AE (2008). Geographic locality and host identity shape fungal endophyte communities in cupressaceous trees. Mycological Research.

[ref-17] Koide RT, Ricks KD, Davis ER (2017). Climate and dispersal influence the structure of leaf fungal endophyte communities of *Quercus gambelii* in the eastern Great Basin, USA. Fungal Ecology.

[ref-18] Kołwzan B, Adamiak W, Rybak J (2011). Sanitary Biology.

[ref-19] Lee N, Yoo H, Yang H (2021). Cluster analysis of medicinal plants and targets based on multipartite network. Biomolecules.

[ref-20] Lee SS, Muhamad A, Tong J (2015). Mangrove guidebook for Malaysia.

[ref-21] Li J, Chen C, Fang T, Wu L, Liu W, Tang J, Long Y (2022). New steroid and isocoumarin from the mangrove endophytic fungus *Talaromyces* sp. SCNU-F0041. Molecules.

[ref-22] Liang Y, Wu W, Li R, Lu Y, Wang G, Tan S, Chen H, Xi J, Huang X, He C, Yi K (2023). Evaluation of *Bacillus subtilis* Czk1 metabolites by LC–MS/MS and their antifungal potential against *Pyrrhoderma noxium* causing brow rot disease. Agriculture.

[ref-23] Ma N, Yin D, Liu Y, Gao Z, Cao Y, Chen T, Huang Z, Jia Q, Wang D (2023). Succession of endophytic fungi and rhizosphere soil fungi and their correlation with secondary metabolites in *Fagopyrum dibotrys*. Frontiers in Microbiology.

[ref-24] Muthukrishnan S, Prakathi P, Sivakumar T, Thiruvengadam M, Jayaprakash B, Baskar V, Rebezov M, Derkho M, Zengin G, Shariati MA (2022). Bioactive components and health potential of endophytic micro-fungal diversity in medicinal plants. Antibiotics.

[ref-25] Ng BF, Ng WL, Lum WM, Yeap SK, Yong YS (2024a). Feasibility of biomarker-based taxonomic classification: a case study of the marine red alga *Laurencia snackeyi* (Weber Bosse) M. Masuda. Phycology.

[ref-26] Ng WL, Tan JK, Gnanaraj C, Shah MD, Nor Rashid N, Abdullah I, Yong YS (2024b). Cytotoxicity of *Physalis minima* Linn (Solanaceae) fruit against HCT116 and HT29 colorectal cancer cell lines. Natural Product Research.

[ref-27] Ogbe AA, Finnie JF, Van Staden J (2020). The role of endophytes in secondary metabolites accumulation in medicinal plants under abiotic stress. South African Journal of Botany.

[ref-28] Otero V, Van De Kerchove R, Satyanarayana B, Mohd-Lokman H, Lucas R, Dahdouh-Guebas F (2019). An analysis of the early regeneration of mangrove forests using landsat time series in the Matang Mangrove Forest Reserve, Peninsular Malaysia. Remote Sensing.

[ref-29] Panchalingam H, Ashfield-Crook N, Naik V, Frenken R, Foster K, Tomlin R, Shapcott A, Kurtböke DI (2022a). Testing the biocontrol ability of a *Trichoderma*-Streptomycetes consortium against *Pyrrhoderma noxium* (Corner) LW Zhou and YC Dai in soil. Journal of Fungi.

[ref-30] Panchalingam H, Powell D, Adra C, Foster K, Tomlin R, Quigley BL, Nyari S, Hayes RA, Shapcott A, Kurtböke DI (2022b). Assessing the various antagonistic mechanisms of *Trichoderma* strains against the brown root rot pathogen *Pyrrhoderma noxium* infecting heritage fig trees. Journal of Fungi.

[ref-31] Pence HE, Williams A (2010). ChemSpider: an online chemical information resource. Journal of Chemical Education.

[ref-32] Poveda J, Eugui D, Abril-Urías P, Velasco P (2021). Endophytic fungi as direct plant growth promoters for sustainable agricultural production. Symbiosis.

[ref-33] Raja HA, Miller AN, Pearce CJ, Oberlies NH (2017). Fungal identification using molecular tools: a primer for the natural products research community. Journal of Natural Products.

[ref-34] Saitou N, Nei M (1987). The neighbor-joining method: a new method for reconstructing phylogenetic trees. Molecular Biology and Evolution.

[ref-35] Sargent M (2013). Guide to achieving reliable quantitative LC-MS measurements.

[ref-36] Shi T, Su D, Liu T, Tang K, Camp DG, Qian WJ, Smith RD (2012). Advancing the sensitivity of selected reaction monitoring-based targeted quantitative proteomics. Proteomics.

[ref-37] Singh VK, Kumar A (2023). Secondary metabolites from endophytic fungi: production, methods of analysis, and diverse pharmaceutical potential. Symbiosis.

[ref-38] Sopalun K, Laosripaiboon W, Wachirachaikarn A, Iamtham S (2021). Biological potential and chemical composition of bioactive compounds from endophytic fungi associated with thai mangrove plants. South African Journal of Botany.

[ref-39] Summer LW, Amberg A, Barrett D, Beale MH, Beger R, Daykin CA, Fan TWM, Fiehn O, Goodacre R, Griffin JL, Hankemeier T, Hardy N, Harnly J, Higashi R, Kopka J, Lane AN, Lindon JC, Marriott P, Nicholls AW, Reily MD, Thaden JJ, Viant MR (2007). Proposed minimum reporting standards for chemical analysis. Metabolomics.

[ref-40] Supriadi S, Adhia EM, Wahyuno D, Rahayuningsih S, Karyani N, Dahsyat M (2004). Brown root rot disease of cashew in West Nusa Tenggara: distribution and its causal organism. Indonesian Journal of Agricultural Science.

[ref-41] Survarachakan S, Prasad PJR, Naseem R, de Frutos JP, Kumar RP, Langø T, Cheikh FA, Elle OJ, Lindseth F (2022). Deep learning for image-based liver analysis—a comprehensive review focusing on malignant lesions. Artificial Intelligence in Medicine.

[ref-42] Tamura K, Nei M, Kumar S (2004). Prospects for inferring very large phylogenies by using the neighbor-joining method. Proceedings of the National Academy of Sciences of the United States of America.

[ref-43] Tamura K, Stecher G, Kumar S (2021). MEGA11: molecular evolutionary genetics analysis version 11. Molecular Biology and Evolution.

[ref-44] Teoh WY, Yong YS, Razali FN, Stephenie S, Dawood Shah M, Tan JK, Gnanaraj C, Mohd Esa N (2023). LC-MS/MS and GC-MS analysis for the identification of bioactive metabolites responsible for the antioxidant and antibacterial activities of *Lygodium microphyllum* (Cav.) R. Br. Separations.

[ref-45] Thompson JD, Higgins DG, Gibson TJ (1994). CLUSTAL W: improving the sensitivity of progressive multiple sequence alignment through sequence weighting, position-specific gap penalties and weight matrix choice. Nucleic Acids Research.

[ref-46] Vingataramin L, Frost EH (2015). A single protocol for extraction of gDNA from bacteria and yeast. Biotechniques.

[ref-47] Voordeckers K, De Maeyer D, van der Zande E, Vinces MD, Meert W, Cloots L, Ryan O, Marchal K, Verstrepen KJ (2012). Identification of a complex genetic network underlying *Saccharomyces cerevisiae* colony morphology. Molecular Microbiology.

[ref-48] Wang J-W, Liu D-Y, Zhang H-Z, Tan Z, Zheng C-J, Chen G-Y, Nong X-H (2023). Drimane-type sesquiterpenoids and their anti-inflammatory evaluation from *Pyrrhoderma noxium* HNNU0524. Natural Product Research.

[ref-49] Wu AH, French D (2013). Implementation of liquid chromatography/mass spectrometry into the clinical laboratory. Clinica Chimica Acta.

[ref-50] Yong YS, Chong ETJ, Chen HC, Lee PC, Ling YS (2017). A comparative study of pentafluorophenyl and octadecylsilane columns in high-throughput profiling of biological fluids. Journal of the Chinese Chemical Society.

[ref-51] Zhang L, Niaz SI, Khan D, Wang Z, Zhu Y, Zhou H, Lin Y, Li J, Liu L (2017). Induction of diverse bioactive secondary metabolites from the mangrove endophytic fungus *Trichoderma* sp.(strain 307) by co-cultivation with *Acinetobacter johnsonii* (strain B2). Marine Drugs.

