# Supplementary material for: A double agent? Unveiling the chemical profile of the pathogenic fungus Pyrrhoderma noxium as an endophyte in true mangroves
Source: PeerJ. 2026 Feb 20;14:e20826. doi: 10.7717/peerj.20826 (PMC12927600; doi:10.7717/peerj.20826)
Supplement: Supplemental Information 1 — The compounds detected by LC-MS/MS in all three Pyrrhoderma noxium [file peerj-14-20826-s001.docx]

**Supplementary A. Compound List of *Pyrrhoderma noxium*.**

| No. | Compound Name | *m/z* | mass error (ppm) | Formula | Compound Class | *Pyrrhoderma noxium* | | |
| --- | --- | --- | --- | --- | --- | --- | --- | --- |
|  |  |  |  |  |  | *AA2AA* | *BG3BA* | *SA2AA* |
| 1 | 1-Methyl-2-undecyl-4(1H)-quinolone | 314.2474 | -1.77 | C_21_H_31_NO | Alkaloid | / | / | / |
| 2 | 2,3,4,9-Tetrahydro-1H-β-carboline-3-carboxylic acid | 217.0969 | -1.17 | C_12_H_12_N_2_O_2_ | Alkaloid |  | / |  |
| 3 | 2,4-Quinolinediol | 162.0547 | -1.76 | C_9_H_7_NO_2_ | Alkaloid | / | / | / |
| 4 | 2,8-Diamino-9-pentofuranosyl-3,9-dihydro-6H-purin-6-one | 299.1093 | -1.91 | C_10_H_14_N_6_O_5_ | Alkaloid | / | / | / |
| 5 | 2-Amino-2'-deoxyadenosine | 267.1197 | -1.34 | C_10_H_14_N_6_O_3_ | Alkaloid |  |  | / |
| 6 | 2'-Deoxyadenosine | 252.1087 | -1.70 | C_10_H_13_N_5_O_3_ | Alkaloid | / | / | / |
| 7 | 2-Hydroxyquinoline | 146.0598 | -1.66 | C_9_H_7_NO | Alkaloid |  | / |  |
| 8 | 3-(2-Hydroxyethyl)indole | 162.0910 | -1.47 | C_10_H_11_NO | Alkaloid |  |  | / |
| 9 | 3-(3-Methylbut-2-en-1-yl)-3H-purin-6-amine | 204.1242 | -1.10 | C_10_H_13_N_5_ | Alkaloid | / |  | / |
| 10 | 3-(4-Aminobutyl)hexahydropyrrolo[1,2-a]pyrazine-1,4-dione | 226.1547 | -1.51 | C_11_H_19_N_3_O_2_ | Alkaloid | / | / | / |
| 11 | 3-(5-Isobutyl-3,6-dioxo-2-piperazinyl)propanoic acid | 243.1337 | -0.71 | C_11_H_18_N_2_O_4_ | Alkaloid |  | / | / |
| 12 | 3-(Propan-2-yl)-octahydropyrrolo[1,2-a]pyrazine-1,4-dione | 197.1282 | -1.05 | C_10_H_16_N_2_O_2_ | Alkaloid | / | / | / |
| 13 | 3,5-Di(5-hexenyl)pyrrolizidine | 276.2680 | -1.78 | C_19_H_33_N | Alkaloid |  | / | / |
| 14 | 3-Hydroxy-2-methylpyridine | 110.0603 | 1.54 | C_6_H_7_NO | Alkaloid | / | / | / |
| 15 | 3-Hydroxypicolinic acid | 140.0340 | -1.27 | C_6_H_5_NO_3_ | Alkaloid | / |  |  |
| 16 | 3-Indoleacrylic acid | 188.0703 | -1.00 | C_11_H_9_NO_2_ | Alkaloid | / | / | / |
| 17 | 3-sec-Butylhexahydropyrrolo[1,2-a]pyrazine-1,4-dione | 211.1436 | -1.76 | C_11_H_18_N_2_O_2_ | Alkaloid | / | / | / |
| 18 | 4-(2,3,4-Trihydroxybutyl)-2,6-piperidinedione | 218.1021 | -1.39 | C_9_H_15_NO_5_ | Alkaloid | / | / | / |
| 19 | 4-(4-Ethylbenzyl)-1,2,5-trimethyl-4-piperidinol | 262.2159 | -1.82 | C_17_H_27_NO | Alkaloid |  |  | / |
| 20 | 4-Indolecarbaldehyde | 146.0598 | -1.65 | C_9_H_7_NO | Alkaloid | / | / | / |
| 21 | 4-Methyl-5-thiazoleethanol | 144.0476 | -1.08 | C_6_H_9_NOS | Alkaloid | / | / | / |
| 22 | 5-Methoxyindoleacetic acid | 206.0809 | -1.05 | C_11_H_11_NO_3_ | Alkaloid | / | / | / |
| 23 | 5-Methylcytosine | 126.0662 | -0.40 | C_5_H_7_N_3_O | Alkaloid | / | / | / |
| 24 | 6-Amino-3-ethyl-1-propyl-2,4(1H,3H)-pyrimidinedione | 198.1234 | -1.63 | C_9_H_15_N_3_O_2_ | Alkaloid |  |  | / |
| 25 | 6-Methylquinoline | 144.0807 | -0.61 | C_10_H_9_N | Alkaloid | / | / |  |
| 26 | 7-[2-(3-Hydroxyoctyl)-5-oxo-1-pyrrolidinyl]heptanoic acid | 342.2635 | -1.16 | C_19_H_35_NO_4_ | Alkaloid | / |  |  |
| 27 | 8-Hydroxyquinoline | 146.0598 | -1.70 | C_9_H_7_NO | Alkaloid | / | / | / |
| 28 | Adenine | 136.0616 | -0.91 | C_5_H_5_N_5_ | Alkaloid | / | / | / |
| 29 | Adenosine | 268.1036 | -1.52 | C_10_H_13_N_5_O_4_ | Alkaloid | / | / | / |
| 30 | Atropine | 290.1744 | -1.89 | C_17_H_23_NO_3_ | Alkaloid | / |  | / |
| 31 | Betaine | 118.0864 | 0.91 | C_5_H_11_NO_2_ | Alkaloid |  | / | / |
| 32 | Choline | 104.1071 | 0.90 | C_5_H_13_NO | Alkaloid | / |  | / |
| 33 | Cyclo(L-Leucyl-L-Leucyl-L-Leucyl-L-Leucyl-L-Leucyl) | 566.4271 | -1.07 | C_11_H_18_N_2_O_2_ | Alkaloid | / | / | / |
| 34 | Cyclo(L-Phenylalanyl-L-Prolyl) | 245.1280 | -1.62 | C_14_H_16_N_2_O_2_ | Alkaloid | / | / | / |
| 35 | Cytarabine | 244.0923 | -1.82 | C_9_H_13_N_3_O_5_ | Alkaloid | / |  | / |
| 36 | Cytosine | 112.0506 | -0.71 | C_4_H_5_N_3_O | Alkaloid | / | / | / |
| 37 | Guanine | 152.0565 | -0.91 | C_5_H_5_N_5_O | Alkaloid | / | / |  |
| 38 | Hypoxanthine | 137.0456 | -1.53 | C_5_H_4_N_4_O | Alkaloid | / | / |  |
| 39 | Indole | 118.0653 | 0.07 | C_8_H_7_N | Alkaloid |  | / | / |
| 40 | Isoquinoline | 130.0651 | -0.01 | C_9_H_7_N | Alkaloid |  | / |  |
| 41 | Ligustrazine | 137.1073 | -0.73 | C_8_H_12_N_2_ | Alkaloid | / | / | / |
| 42 | Methylthioadenosine | 298.0963 | -1.96 | C_11_H_15_N_5_O_3_S | Alkaloid | / | / | / |
| 43 | N,N-dimethyl-9H-purin-6-amine | 164.0929 | -0.78 | C_7_H_9_N_5_ | Alkaloid | / | / | / |
| 44 | N-Acetyltyramine | 180.1018 | -1.06 | C_10_H_13_NO_2_ | Alkaloid | / |  | / |
| 45 | Neosaxitoxin | 316.1360 | -1.23 | C_10_H_17_N_7_O_5_ | Alkaloid |  |  | / |
| 46 | Norharman | 169.0758 | -1.15 | C_11_H_8_N_2_ | Alkaloid | / | / | / |
| 47 | Pancracine | 288.1227 | -1.07 | C_16_H_17_NO_4_ | Alkaloid | / | / | / |
| 48 | Pipecolic acid | 130.0863 | 0.03 | C_6_H_11_NO_2_ | Alkaloid | / | / | / |
| 49 | Purine | 121.0509 | 0.59 | C_5_H_4_N_4_ | Alkaloid | / |  | / |
| 50 | Thymine | 127.0502 | 0.04 | C_5_H_6_N_2_O_2_ | Alkaloid | / | / | / |
| 51 | trans-3-Hydroxycotinine | 193.0970 | -0.84 | C_10_H_12_N_2_O_2_ | Alkaloid | / | / | / |
| 52 | trans-Zeatin | 220.1190 | -1.53 | C_10_H_13_N_5_O | Alkaloid | / | / | / |
| 53 | Trigonelline | 138.0549 | -1.06 | C_7_H_7_NO_2_ | Alkaloid | / |  | / |
| 54 | 1-(2-Hydroxypropyl)-L-histidine | 214.1185 | -0.55 | C_9_H_15_N_3_O_3_ | Amino acid | / |  | / |
| 55 | 2-Aminopimelic acid | 176.0916 | -1.21 | C_7_H_13_NO_4_ | Amino acid |  |  | / |
| 56 | 4-Guanidinobutyric acid | 146.0922 | -1.42 | C_5_H_11_N_3_O_2_ | Amino acid | / | / | / |
| 57 | 5-Acetamido-4-oxohexanoic acid | 188.0914 | -1.71 | C_8_H_13_NO_4_ | Amino acid |  | / |  |
| 58 | 6-Oxo-pipecolinic acid | 144.0654 | -1.11 | C_6_H_9_NO_3_ | Amino acid | / | / | / |
| 59 | Acetyl-L-carnitine | 204.1227 | -1.64 | C_9_H_17_NO_4_ | Amino acid | / | / | / |
| 60 | Afalanine | 208.0968 | -0.68 | C_11_H_13_NO_3_ | Amino acid | / | / | / |
| 61 | Carboxynorspermidine | 176.1391 | -1.45 | C_7_H_17_N_3_O_2_ | Amino acid |  | / |  |
| 62 | Carnitine | 162.1121 | -1.69 | C_7_H_15_NO_3_ | Amino acid |  |  | / |
| 63 | D-(+)-Pipecolinic acid | 130.0863 | 0.02 | C_6_H_11_NO_2_ | Amino acid |  | / |  |
| 64 | D-(+)-Proline | 116.0708 | 1.24 | C_5_H_9_NO_2_ | Amino acid |  | / |  |
| 65 | D-(+)-Pyroglutamic acid | 130.0498 | -0.57 | C_5_H_7_NO_3_ | Amino acid | / | / | / |
| 66 | Decanoylcarnitine | 316.2477 | -1.74 | C_17_H_33_NO_4_ | Amino acid |  | / | / |
| 67 | D-lysopine | 219.1336 | -1.71 | C_9_H_18_N_2_O_4_ | Amino acid | / | / | / |
| 68 | Isoleucine | 132.1018 | -1.10 | C_6_H_13_NO_2_ | Amino acid |  | / |  |
| 69 | L-Ergothioneine | 230.0955 | -1.35 | C_9_H_15_N_3_O_2_S | Amino acid | / |  | / |
| 70 | L-Histidine | 156.0765 | -1.63 | C_6_H_9_N_3_O_2_ | Amino acid |  | / | / |
| 71 | L-Norleucine | 132.1018 | -1.08 | C_6_H_13_NO_2_ | Amino acid | / | / | / |
| 72 | L-Phenylalanine | 166.0861 | -1.05 | C_9_H_11_NO_2_ | Amino acid | / | / | / |
| 73 | L-Pyroglutamic acid | 130.0498 | -0.86 | C_5_H_7_NO_3_ | Amino acid |  |  | / |
| 74 | L-Tyrosine | 182.0810 | -1.08 | C_9_H_11_NO_3_ | Amino acid | / |  |  |
| 75 | N(2)-succinyl-L-glutamic acid | 248.0761 | -1.44 | C_9_H_13_NO_7_ | Amino acid | / | / |  |
| 76 | N-(Isopropylcarbamoyl)-L-leucine | 217.1545 | -0.94 | C_10_H_20_N_2_O_3_ | Amino acid | / |  | / |
| 77 | N3,N4-Dimethyl-L-arginine | 203.1501 | -1.29 | C_8_H_18_N_4_O_2_ | Amino acid | / | / | / |
| 78 | N6-Acetyl-L-lysine | 189.1232 | -0.74 | C_8_H_16_N_2_O_3_ | Amino acid | / |  |  |
| 79 | N-Acetyl-L-leucine | 174.1122 | -1.43 | C_8_H_15_NO_3_ | Amino acid | / |  | / |
| 80 | O-(3-Carboxypropanoyl)homoserine | 220.0813 | -1.42 | C_8_H_13_NO_6_ | Amino acid |  |  | / |
| 81 | Propionylcarnitine | 218.1384 | -1.13 | C_10_H_19_NO_4_ | Amino acid | / | / | / |
| 82 | Tilarginine | 189.1344 | -1.30 | C_7_H_16_N_4_O_2_ | Amino acid | / | / | / |
| 83 | Tryptophan | 205.0968 | -1.50 | C_11_H_12_N_2_O_2_ | Amino acid |  | / |  |
| 84 | Urocanic acid | 139.0501 | -1.28 | C_6_H_6_N_2_O_2_ | Amino acid | / | / | / |
| 85 | (2-Aminophenyl)methanol | 124.0757 | -0.18 | C_7_H_9_NO | Aromatic |  |  | / |
| 86 | (4E)-1,7-Diphenyl-4-hepten-3-one | 265.1582 | -1.73 | C_19_H_20_O | Aromatic |  | / |  |
| 87 | (5-Benzyl-1,3-dimethyl-4-stearoyl-1H-pyrrol-2-yl)acetic acid | 510.3935 | -1.21 | C_33_H_51_NO_3_ | Aromatic | / | / |  |
| 88 | 2-(2-Amino-3-methylbutanamido)-3-phenylpropanoic acid | 265.1543 | -1.69 | C_14_H_20_N_2_O_3_ | Aromatic |  | / |  |
| 89 | 2-Aminobenzimidazole | 134.0712 | -0.80 | C_7_H_7_N_3_ | Aromatic |  | / |  |
| 90 | 3,3,5-Trimethyl-6-oxo-2-phenyl-3,6-dihydro-2H-pyran-4-yl pivalate | 317.1741 | -2.00 | C_19_H_24_O_4_ | Aromatic |  | / |  |
| 91 | 4-Phenylbutyric acid | 165.0908 | -1.23 | C_10_H_12_O_2_ | Aromatic |  | / |  |
| 92 | Acetanisole | 151.0754 | 0.90 | C_9_H_10_O_2_ | Aromatic |  | / | / |
| 93 | Benzyl-[2-(2-ethyl-2-methyl-4-p-tolyl-tetrahydro-pyran-4-yl)-ethyl]-amine | 352.2631 | -1.65 | C_24_H_33_NO | Aromatic | / | / | / |
| 94 | Benzylideneacetone | 147.0803 | -1.34 | C_10_H_10_O | Aromatic |  |  | / |
| 95 | Carbidopa | 227.1024 | -0.96 | C_10_H_14_N_2_O_4_ | Aromatic | / | / | / |
| 96 | Kynurenic acid | 190.0494 | -1.91 | C_10_H_7_NO_3_ | Aromatic | / | / | / |
| 97 | N-[3-(2,2-Dimethyltetrahydro-2H-pyran-4-yl)-3-phenylpropyl]-N-(4-methoxybenzyl)acetamide | 410.2681 | -1.36 | C_26_H_35_NO_3_ | Aromatic |  | / | / |
| 98 | Nonivamide | 294.2060 | -1.61 | C_17_H_27_NO_3_ | Aromatic |  |  | / |
| 99 | Phenazone | 189.1020 | -1.41 | C_11_H_12_N_2_O | Aromatic |  | / |  |
| 100 | Phenyl phosphate | 175.0153 | -1.56 | C_6_H_7_O_4_P | Aromatic | / | / | / |
| 101 | {(1R,2R,5R)-2-[(2R,3E,5R)-5,6-Dimethyl-3-hepten-2-yl]-5-[(1aS,4aS,7S,8aR)-7-hydroxy-4a-methyl-4-oxo-4,4a,5,6,7,8-hexahydro-1aH-naphtho[1,8a-b]oxiren-3-yl]-1-methylcyclopentyl}acetaldehyde | 443.3148 | -1.60 | C_28_H_42_O_4_ | Cyclic ketone | / |  |  |
| 102 | Isophorone | 139.1116 | -1.04 | C_9_H_14_O | Cyclic ketone | / | / | / |
| 103 | (2S)-1-Hydroxy-3-[(9Z)-9-octadecenoyloxy]-2-propanyl nonadecanoate | 637.5761 | -0.48 | C_40_H_76_O_5_ | Ester | / | / | / |
| 104 | (9Z,11R,12Z,15Z)-11-(Nonadecanoyloxy)-9,12,15-octadecatrienoic acid | 575.5025 | -1.16 | C_37_H_66_O_4_ | Ester |  | / | / |
| 105 | [(7E,9Z)-Dodeca-7,9-dienyl] acetate | 225.1846 | -1.30 | C_14_H_24_O_2_ | Ester |  | / | / |
| 106 | 1-Acetoxy-3-(palmitoyloxy)-2-propanyl (9Z,12Z,15Z)-9,12,15-octadecatrienoate | 633.5067 | -1.22 | C_39_H_68_O_6_ | Ester | / |  |  |
| 107 | Dihomo-γ-linolenic acid ethyl ester | 335.2939 | -1.94 | C_22_H_38_O_2_ | Ester |  | / | / |
| 108 | Methyl N-{(2R)-2-[2-(hydroxyamino)-2-oxoethyl]heptanoyl}glycinate | 275.1598 | -1.58 | C_12_H_22_N_2_O_5_ | Ester | / | / |  |
| 109 | (11E)-9-Hydroxy-13-oxo-11-tridecenoic acid | 243.1589 | -0.94 | C_13_H_22_O_4_ | Fatty acid |  |  | / |
| 110 | (2E)-Decenoic acid | 171.1378 | -1.22 | C_10_H_18_O_2_ | Fatty acid | / | / | / |
| 111 | (4E,6E)-2-(Hydroxymethyl)-3-(3-methoxy-3-oxopropyl)-4,6-decadienoic acid | 285.1691 | -1.84 | C_15_H_24_O_5_ | Fatty acid | / | / |  |
| 112 | 12-{[(3s,5s,7s)-Adamantan-1-ylcarbamoyl]amino}dodecanoic acid | 393.3103 | -1.61 | C_23_H_40_N_2_O_3_ | Fatty acid |  | / | / |
| 113 | 2E,6E-decadienoic acid | 169.1221 | -1.22 | C_10_H_16_O_2_ | Fatty acid |  | / |  |
| 114 | 3-[(1E,3E)-1,3-Heptadien-1-yl]pentanedioic acid | 227.1274 | -1.69 | C_12_H_18_O_4_ | Fatty acid | / |  | / |
| 115 | 3-Methoxydocosanoic acid | 371.3514 | -1.68 | C_23_H_46_O_3_ | Fatty acid | / |  |  |
| 116 | 5-({2-[(tert-Butoxycarbonyl)amino]hexadecyl}amino)pentanoic acid | 457.3991 | -1.95 | C_26_H_52_N_2_O_4_ | Fatty acid | / | / | / |
| 117 | 9-(Methoxycarbonyl)-9-decenoic acid | 211.1326 | -1.31 | C_12_H_20_O_4_ | Fatty acid |  | / |  |
| 118 | 9S,13R-12-Oxophytodienoic acid | 293.2105 | -2.00 | C_18_H_28_O_3_ | Fatty acid | / |  |  |
| 119 | (2E,4E)-N-(1-Hydroxy-3-methyl-2-pentanyl)-2-methyl-2,4-hexadienamide | 226.1797 | -2.00 | C_13_H_23_NO_2_ | Fatty amide | / |  | / |
| 120 | (2R)-N-[(2S,3R,4E,8E)-1,3-Dihydroxy-9-methyl-4,8-octadecadien-2-yl]-2-hydroxypentadecanamide | 552.4978 | -1.69 | C_34_H_65_NO_4_ | Fatty amide | / |  | / |
| 121 | (2S,3R,4S)-2-(Hydroxymethyl)-4-[(11S)-11-hydroxy-13-methyltetradecyl]-3-azetidinol | 330.2997 | -1.61 | C_19_H_39_NO_3_ | Fatty amide |  | / | / |
| 122 | (3R,4R,6E)-2-Amino-2-(hydroxymethyl)-6-icosene-1,3,4,14-tetrol | 390.3208 | -1.50 | C_21_H_43_NO_5_ | Fatty amide |  | / | / |
| 123 | 3-Hydroxy-N-[(3S)-2-oxotetrahydro-3-furanyl]octanamide | 244.1543 | -0.34 | C_12_H_21_NO_4_ | Fatty amide | / |  |  |
| 124 | 4,4-Dimethoxy-N,N,3-trimethylbutanamide | 190.1436 | -1.14 | C_9_H_19_NO_3_ | Fatty amide | / |  | / |
| 125 | Crucigasterin E | 280.2629 | -1.87 | C_18_H_33_NO | Fatty amide |  | / | / |
| 126 | Myristamide | 228.2318 | -1.56 | C_14_H_29_NO | Fatty amide | / | / | / |
| 127 | N-[(1-Ethyl-2-pyrrolidinyl)methyl]-N'-propylethanediamide | 242.1858 | -1.58 | C_12_H_23_N_3_O_2_ | Fatty amide | / | / | / |
| 128 | N-[(2S,3R,4E,8E,10E)-1,3-Dihydroxy-4,8,10-octadecatrien-2-yl]hexadecanamide | 534.4871 | -1.89 | C_34_H_63_NO_3_ | Fatty amide | / |  |  |
| 129 | Semiplenamide A | 366.3362 | -1.85 | C_23_H_43_NO_2_ | Fatty amide | / |  | / |
| 130 | Stearamide | 284.2941 | -1.88 | C_18_H_37_NO | Fatty amide |  | / | / |
| 131 | (8E,10Z)-8,10-Tetradecadienal | 209.1899 | -0.35 | C_14_H_24_O | Fatty carbonyl | / |  | / |
| 132 | (8Z,11Z,14Z)-Heptadecatrienal | 249.2208 | -1.94 | C_17_H_28_O | Fatty carbonyl | / | / |  |
| 133 | Sulcatone | 127.1117 | -0.14 | C_8_H_14_O | Fatty carbonyl | / | / |  |
| 134 | (2S)-2-{[8-(1,2,3,4,4a,4b,7,8,8a,8b-Decahydro-2-biphenylenyl)octyl]oxy}-3-hydroxypropyl palmitate | 603.5349 | 0.37 | C_39_H_70_O_4_ | Glycerolipid | / |  |  |
| 135 | 1-[(9Z)-Tetradecenoyl]-2-[(5Z,8Z,11Z,14Z)-icosatetraenoyl]-sn-glycerol | 587.4665 | -0.99 | C_37_H_62_O_5_ | Glycerolipid | / | / | / |
| 136 | 1-Hexadecyl-2-amino-2-deoxy-sn-glycerol | 316.3205 | -1.72 | C_19_H_41_NO_2_ | Glycerolipid |  |  | / |
| 137 | 1-Linoleoyl-2-myristoyl-sn-glycerol | 565.4818 | -1.25 | C_35_H_64_O_5_ | Glycerolipid | / |  | / |
| 138 | 1-Myristoyl-2-docosapentaenoyl-sn-glycerol | 615.4979 | -0.85 | C_39_H_66_O_5_ | Glycerolipid | / | / | / |
| 139 | 1-α-Linolenoyl-2-palmitoleoyl-sn-glycerol | 589.4821 | -1.61 | C_37_H_64_O_5_ | Glycerolipid | / | / | / |
| 140 | Maltol | 127.0390 | 0.11 | C_6_H_6_O_3_ | Heterocyclic |  | / | / |
| 141 | 5-(6-hydroxy-6-methyloctyl)-2(5H)-furan-2-one | 227.1638 | -1.50 | C_13_H_22_O_3_ | Lactone | / | / | / |
| 142 | 5-(7-Hydroxy-6-methyloctyl)-2(5H)-furanone | 227.1638 | -1.67 | C_13_H_22_O_3_ | Lactone | / | / | / |
| 143 | 6-Amyl-2-pyrone | 167.1064 | -1.36 | C_10_H_14_O_2_ | Lactone |  | / |  |
| 144 | Loliolide | 197.1170 | -1.26 | C_11_H_16_O_3_ | Lactone |  |  | / |
| 145 | N-(3-Oxodecanoyl)-L-homoserine lactone | 270.1695 | -1.93 | C_14_H_23_NO_4_ | Lactone | / |  |  |
| 146 | N-tetradecanoyl-L-Homoserine Lactone | 312.2527 | -1.94 | C_18_H_33_NO_3_ | Lactone | / |  |  |
| 147 | Sedanolide | 195.1376 | -1.70 | C_12_H_18_O_2_ | Lactone | / | / | / |
| 148 | (2S)-2-[(2-{[(2S,3R,4R,5S,6R)-3-Acetamido-2,5-dihydroxy-6-(hydroxymethyl)tetrahydro-2H-pyran-4-yl]oxy}propyl)amino]propanoic acid | 351.1756 | -1.93 | C_14_H_26_N_2_O_8_ | Monosaccharide | / |  | / |
| 149 | 2-Acetamidoglucal | 204.0864 | -1.17 | C_8_H_13_NO_5_ | Monosaccharide |  |  | / |
| 150 | 4,6-dideoxy-4-(3-deoxy-L-glycero-tetronamido)-2-O-methyl-α-D-mannopyranose | 280.1385 | -1.87 | C_11_H_21_NO_7_ | Monosaccharide | / | / | / |
| 151 | N-Propionylhexopyranosylamine | 236.1125 | -1.18 | C_9_H_17_NO_6_ | Monosaccharide | / |  | / |
| 152 | Alanylleucine | 203.1386 | -1.73 | C_9_H_18_N_2_O_3_ | Peptide | / | / | / |
| 153 | Glycyl-L-leucine | 189.1230 | -1.79 | C_8_H_16_N_2_O_3_ | Peptide |  | / |  |
| 154 | L-Alanyl-L-proline | 187.1075 | -1.43 | C_8_H_14_N_2_O_3_ | Peptide |  | / |  |
| 155 | L-gamma-Glutamyl-L-leucine | 261.1441 | -1.52 | C_13_H_25_N_5_O_4_ | Peptide |  | / |  |
| 156 | L-Leucyl-(4R)-4-hydroxy-L-proline | 245.1491 | -1.82 | C_11_H_20_N_2_O_4_ | Peptide |  | / |  |
| 157 | L-Leucyl-L-valine | 231.1702 | -1.38 | C_11_H_22_N_2_O_3_ | Peptide |  | / |  |
| 158 | Lysyl-Leucine | 260.1964 | -2.00 | C_12_H_25_N_3_O_3_ | Peptide |  | / |  |
| 159 | N-{[(2-Methyl-2-propanyl)oxy]carbonyl}leucylleucinamide | 344.2539 | -1.63 | C_17_H_33_N_3_O_4_ | Peptide | / |  | / |
| 160 | Prolyl-hydroxyproline | 229.1181 | -0.82 | C_10_H_16_N_2_O_4_ | Peptide | / | / |  |
| 161 | 2-[(2E)-3,7-Dimethyl-2,6-octadien-1-yl]phenol | 231.1741 | -1.11 | C_16_H_22_O | Phenolic | / |  |  |
| 162 | 8-Gingerol | 323.2210 | -1.86 | C_19_H_30_O_4_ | Phenolic | / | / | / |
| 163 | trans-Clovamide | 360.1075 | -0.82 | C_18_H_17_NO_7_ | Phenolic |  | / |  |
| 164 | trans-p-Coumaraldehyde | 149.0596 | -1.06 | C_9_H_8_O_2_ | Phenolic | / | / | / |
| 165 | (2R)-1-[(9Z)-9-Hexadecenoyloxy]-3-(phosphonooxy)-2-propanyl (9Z)-9-octadecenoate | 690.5064 | -1.06 | C_37_H_69_O_8_P | Phospholipid | / | / | / |
| 166 | (2R)-3-{[(2-Aminoethoxy)(hydroxy)phosphoryl]oxy}-2-[(9Z)-9-tetradecenoyloxy]propyl palmitate | 662.4745 | -1.97 | C_35_H_68_NO_8_P | Phospholipid | / |  | / |
| 167 | (2R)-3-{[(2-Aminoethoxy)(hydroxy)phosphoryl]oxy}-2-hydroxypropyl (9Z)-9-hexadecenoate | 452.2763 | -1.81 | C_21_H_42_NO_7_P | Phospholipid | / |  | / |
| 168 | 1-Linoleoyl-sn-glycero-3-phospho-D-myo-inositol | 597.3028 | -1.09 | C_27_H_49_O_12_P | Phospholipid | / | / | / |
| 169 | 1-Palmitoleoyl-2-arachidonoyl-sn-glycero-3-phosphoethanolamine | 738.5066 | -0.32 | C_41_H_72_NO_8_P | Phospholipid | / |  | / |
| 170 | Glycerophospho-N-palmitoyl ethanolamine | 454.2919 | -1.69 | C_21_H_44_NO_7_P | Phospholipid | / | / | / |
| 171 | O-(Hydroxy{(2R)-2-hydroxy-3-[(9Z,12Z)-9,12-octadecadienoyloxy]propoxy}phosphoryl)-L-serine | 522.2820 | -1.29 | C_24_H_44_NO_9_P | Phospholipid | / | / | / |
| 172 | (5S)-5-Methyl-3-{13-[(2R,2'R,5S,5'S)-5'-undecyloctahydro-2,2'-bifuran-5-yl]tridecyl}-2(5H)-furanone | 575.5025 | -1.54 | C_37_H_66_O_4_ | Polyketide |  |  | / |
| 173 | C16-Dihydroceramide | 540.5343 | -1.43 | C_34_H_69_NO_3_ | Sphingolipid | / |  | / |
| 174 | N-Palmityl-octadeca-sphinga-4,8-dienine | 536.5027 | -1.78 | C_34_H_65_NO_3_ | Sphingolipid | / | / | / |
| 175 | (3beta,5alpha,22E)-3,5,9-Trihydroxyergosta-7,22-dien-6-one | 445.3305 | -1.64 | C_28_H_44_O_4_ | Steroid | / | / | / |
| 176 | Brassicasterol | 395.3302 | -1.92 | C_28_H_42_O | Steroid | / | / | / |
| 177 | Cholesta-5,7,22-trien-3-β-ol | 383.3302 | -1.31 | C_27_H_42_O | Steroid |  | / | / |
| 178 | N-Butyl-10-[(7alpha,17beta)-17-hydroxy-3-oxoandrostan-7-yl]-N-methyldecanamide | 530.4560 | -1.47 | C_34_H_59_NO_3_ | Steroid |  | / |  |
| 179 | Solanidine | 398.3412 | -1.84 | C_27_H_43_NO | Steroid | / | / | / |
| 180 | (-)-Camphor | 153.1271 | -1.56 | C_10_H_16_O | Terpenoid |  | / | / |
| 181 | (-)-Caryophyllene oxide | 221.1897 | -1.44 | C_15_H_24_O | Terpenoid | / | / | / |
| 182 | (+)-ar-Turmerone | 217.1583 | -1.72 | C_15_H_20_O | Terpenoid | / | / | / |
| 183 | (2E,6Z)-13-Hydroxy-1-(2-hydroxy-5-methoxy-3-methylphenyl)-3,7,11,15-tetramethyl-2,6,14-hexadecatriene-5,12-dione | 457.2942 | -1.45 | C_28_H_40_O_5_ | Terpenoid |  | / |  |
| 184 | (9E,15E)-21-(5,5-Dimethyltetrahydro-2-furanyl)-10,18,20-trihydroxy-3,5,7,11,13,17,19-heptamethyl-9,15-henicosadien-8-one | 551.4663 | -1.10 | C_34_H_62_O_5_ | Terpenoid |  | / |  |
| 185 | (all-E)-2-(3,7-Dimethyl-9-(2,6,6-trimethyl-1-cyclohexen-1-yl)-2,4,6,8-nonatetraenylidene)-5,5- dimethyl-1,3-cyclohexanedione | 407.2938 | -1.71 | C_28_H_38_O_2_ | Terpenoid | / |  | / |
| 186 | 3B,9a,11-Trihydroxy-6-oxodrim-7-ene | 269.1742 | -1.98 | C_15_H_24_O_4_ | Terpenoid | / |  | / |
| 187 | 3-Hydroxyolean-12-en-11-one | 441.3721 | -1.36 | C_30_H_48_O_2_ | Terpenoid | / | / | / |
| 188 | 3-Methoxy-4-hydroxy-5-all-trans-heptaprenylbenzoic acid | 645.4866 | -1.81 | C_43_H_64_O_4_ | Terpenoid |  |  | / |
| 189 | Carvone | 151.1116 | -1.37 | C_10_H_14_O | Terpenoid | / | / | / |
| 190 | Linalyl propionate | 211.1690 | -1.19 | C_13_H_22_O_2_ | Terpenoid |  |  | / |
| 191 | Parthenolide | 249.1481 | -1.92 | C_15_H_20_O_3_ | Terpenoid | / | / |  |
| 192 | Pulegone | 153.1271 | -1.64 | C_10_H_16_O | Terpenoid | / | / | / |
| 193 | β-Ionone | 193.1584 | -1.81 | C_13_H_20_O | Terpenoid | / | / | / |
| 194 | 6-Hydroxynicotinic acid | 140.0341 | -1.08 | C_6_H_5_NO_3_ | Vitamin B | / | / | / |
| 195 | Nicotinamide | 123.0553 | 0.39 | C_6_H_6_N_2_O | Vitamin B | / | / | / |
| 196 | Nicotinic acid | 124.0394 | 0.33 | C_6_H_5_NO_2_ | Vitamin B | / | / | / |
| 197 | Pyridoxal | 168.0658 | 0.58 | C_8_H_9_NO_3_ | Vitamin B | / | / | / |
| 198 | Pyridoxine | 170.0809 | -1.48 | C_8_H_11_NO_3_ | Vitamin B | / | / | / |
| 199 | Thiamine | 265.1113 | -1.74 | C_12_H_16_N_4_OS | Vitamin B |  |  | / |

*Note:* “/” indicates the presence of compound in the LC-MS/MS analyses.
