# Supplementary material for: A double agent? Unveiling the chemical profile of the pathogenic fungus Pyrrhoderma noxium as an endophyte in true mangroves
Source: PeerJ. 2026 Feb 20;14:e20826. doi: 10.7717/peerj.20826 (PMC12927600; doi:10.7717/peerj.20826)
Supplement: Supplemental Information 2 — number and percentage of compounds in each major compound class [file peerj-14-20826-s002.docx]

| No. | Compound Class | *Pyrrhoderma noxium* | | | | | |
| --- | --- | --- | --- | --- | --- | --- | --- |
|  |  | *AA2AA* | | *BG3BA* | | *SA2AA* | |
|  |  | *N* | *%* | *N* | *%* | *N* | *%* |
| 1 | Other lipids | 34 | 25.76 | 27 | 19.57 | 34 | 23.29 |
| 2 | Other alkaloids | 41 | 31.06 | 39 | 28.26 | 45 | 30.82 |
| 3 | Amino acid and peptides | 22 | 16.67 | 29 | 21.01 | 24 | 16.44 |
| 4 | Aromatics | 5 | 3.79 | 13 | 9.42 | 9 | 6.16 |
| 5 | Terpenoids | 9 | 6.82 | 10 | 7.25 | 11 | 7.53 |
| 6 | Phenolics | 3 | 2.27 | 3 | 2.17 | 2 | 1.37 |
| 7 | Others | 18 | 13.64 | 17 | 12.32 | 21 | 14.38 |
|  | **Total** | 132 | 100.00 | 138 | 100.00 | 146 | 100.00 |

*Note*: “*N*” indicates the number of compounds and “*%*” indicates the percentage of compounds.
