# Supplementary material for: A double agent? Unveiling the chemical profile of the pathogenic fungus Pyrrhoderma noxium as an endophyte in true mangroves
Source: PeerJ. 2026 Feb 20;14:e20826. doi: 10.7717/peerj.20826 (PMC12927600; doi:10.7717/peerj.20826)
Supplement: Supplemental Information 3 — Summary tables for the potential bioactivities of compounds extracted from P. noxium [file peerj-14-20826-s003.docx]

**Supplementary C. Summary Table for the Potential Bioactivities of Compound Extracted from *Pyrrhoderma noxium*.**

| No. | Compound Name | Anticancer | Antioxidant | Anti-inflammatory | Antibacterial | Antifungal | Antiviral |
| --- | --- | --- | --- | --- | --- | --- | --- |
| 1 | (-)-Caryophyllene oxide | / | / | / |  |  |  |
| 2 | (+)-ar-Turmerone |  |  | / | / | / |  |
| 3 | 3-Hydroxy-2-methylpyridine |  |  |  | / | / |  |
| 4 | 3-Indoleacrylic acid |  |  | / |  |  |  |
| 5 | 4-Guanidinobutyric acid |  |  |  | / |  |  |
| 6 | 4-Phenylbutyric acid |  |  | / |  |  |  |
| 7 | 5-Methoxyindoleacetic acid |  | / |  |  |  |  |
| 8 | 6-Amyl-2-pyrone |  |  |  |  | / |  |
| 9 | 8-Gingerol | / | / | / |  |  |  |
| 10 | 8-Hydroxyquinoline | / |  |  | / | / |  |
| 11 | Acetanisole |  |  |  | / |  |  |
| 12 | Acetyl-L-carnitine |  | / | / |  |  |  |
| 13 | Adenosine |  | / |  |  |  |  |
| 14 | Atropine | / |  | / |  |  | / |
| 15 | Benzylideneacetone |  |  |  | / |  |  |
| 16 | Betaine |  | / | / |  |  |  |
| 17 | Brassicasterol | / |  |  |  |  |  |
| 18 | Carbidopa | / | / |  |  |  |  |
| 19 | Carvone |  |  |  |  | / |  |
| 20 | Crucigasterin E |  |  |  | / |  |  |
| 21 | Cyclo(L-Phenylalanyl-L-Prolyl) |  |  |  | / | / |  |
| 22 | Cytarabine | / |  |  |  |  | / |
| 23 | Isoleucine | / | / | / | / |  | / |
| 24 | Kynurenic acid | / | / | / |  |  |  |
| 25 | L-Ergothioneine |  | / | / |  |  |  |
| 26 | Ligustrazine | / | / |  |  |  |  |
| 27 | Loliolide | / | / | / |  |  |  |
| 28 | Maltol | / | / | / |  |  |  |
| 29 | Methylthioadenosine | / | / | / |  |  |  |
| 30 | N-(3-Oxodecanoyl)-L-homoserine lactone |  |  | / |  |  |  |
| 31 | N-Acetyl-L-leucine |  |  | / |  |  |  |
| 32 | N-Acetyltyramine |  |  |  | / |  |  |
| 33 | Neosaxitoxin |  |  | / |  |  |  |
| 34 | Nicotinamide |  | / | / |  |  |  |
| 35 | Nicotinic acid |  | / | / |  |  |  |
| 36 | Nonivamide |  |  | / |  |  |  |
| 37 | Norharman | / |  |  | / |  |  |
| 38 | Pancracine |  |  |  |  |  | / |
| 39 | Parthenolide | / |  | / |  |  |  |
| 40 | Pyridoxine | / |  |  |  |  |  |
| 41 | Sedanolide | / | / |  |  | / |  |
| 42 | Solanidine |  |  | / |  |  |  |
| 43 | Thiamine |  | / |  |  |  |  |
| 44 | trans-Clovamide |  | / | / |  | / | / |
| 45 | Trigonelline |  | / | / | / | / | / |
| 46 | β-Ionone | / |  |  |  | / |  |

*Note:* “/” indicates the presence of potential bioactivities for the compound.
