# Supplementary material for: A double agent? Unveiling the chemical profile of the pathogenic fungus Pyrrhoderma noxium as an endophyte in true mangroves
Source: PeerJ. 2026 Feb 20;14:e20826. doi: 10.7717/peerj.20826 (PMC12927600; doi:10.7717/peerj.20826)
Supplement: Supplemental Information 4 — The literature search were done on the detected compounds on their potential bioactivities and the potential bioactivities [file peerj-14-20826-s004.docx]

**Supplementary D. Detailed Potential Bioactivities of Compounds Extracted from *Pyrrhoderma noxium*.**

Table 1. Cytotoxic metabolites identified from *P. noxium*.

| No. | Type of Cancer | Cell line / Model | Half-maximal inhibitory concentration (IC_50_) | Compound | | References |
| --- | --- | --- | --- | --- | --- | --- |
| 1. | Breast Cancer | MCF-7 | < 10 µg/mL | | (-)-Caryophyllene oxide | (Sultan et al. 2019) |
|  |  |  |  |  | Carbidopa | (Chen et al. 2022) |
|  |  |  | > 10 µg/mL | | β-Ionone | (Dong et al. 2019) |
|  |  | MDA-MB-231 | < 10 µg/mL | | Atropine | (Ahmed et al. 2022) |
|  |  |  | > 10 µg/mL | | Ligustrazine | (Pan et al. 2015) |
|  |  |  | N/A | | Loliolide | (Yang et al. 2023) |
|  |  | T47D | < 10 µg/mL | | Atropine | (Ahmed et al. 2022) |
|  |  |  |  |  | Carbidopa | (Chen et al. 2022) |
| 2. | Liver Cancer | HepJ5 | < 100 µg/mL | | Sedanolide | (Hsieh et al. 2015) |
|  |  | SH-J1 | < 10 µg/mL | | Parthenolide | (Wen et al. 2002) |
|  |  | Liver metastatic model | N/A | | Isoleucine | (Murata & Moriyama 2007) |
|  |  | H22-bearing mice | N/A | | Maltol | (Li et al. 2015) |
| 3. | Blood Cancer | K562 | > 10 µg/mL | | β-Ionone | (Faezizadeh et al. 2016) |
|  |  | THP-1 | > 100 µg/mL | | Pyridoxine | (Yang et al. 2020b) |
|  |  | U937 |  |  |  |  |
|  |  | HL-60/vcr | < 50 µg/mL | | Cytarabine | (Liu et al. 2022) |
|  |  | 32D-FLT3-ITD |  |  |  |  |
|  |  | MOLT-3 |  |  |  |  |
|  |  | TALL-104 |  |  |  |  |
|  |  | J.RT3-T3.5 |  |  |  |  |
|  |  | Jurkat, Clone E6-1 |  |  |  |  |
| 4. | Prostate Cancer | LNCaP | < 10 µg/mL | | Brassicasterol | (Xu et al. 2020) |
|  |  |  | > 10 µg/mL | | Carbidopa | (Chen et al. 2020) |
|  |  |  |  |  | β-Ionone | (Jones et al. 2013) |
|  |  | DU145 | < 50 µg/mL | | β-Ionone | (Jones et al. 2013) |
|  |  |  | > 50 µg/mL | | Atropine | (Yahia et al. 2018) |
|  |  | PC-3 | > 10 µg/mL | | β-Ionone | (Jones et al. 2013) |
|  |  |  | N/A | | Brassicasterol | (Xu et al. 2020) |
|  |  | VCAP | < 50 µg/mL | | Carbidopa | (Chen et al. 2020) |
| 5. | Colorectal Cancer | HCT116 | < 50 µg/mL | | 8-Gingerol | (Hu et al. 2020) |
|  |  |  | N/A | | Loliolide | (Yang et al. 2023) |
|  |  | DLD-1 | > 10 µg/mL | | 8-Gingerol | (Hu et al. 2020) |
|  |  | HT-29 | > 50 µg/mL | | Kynurenic acid | (Walczak et al. 2014b) |
|  |  | SW480 | < 10 µg/mL | | Ligustrazine | (Bian et al. 2021) |
|  |  | CT26 |  |  |  |  |
| 6. | Skin Cancer | B16F10 | 50 µg/mL | | Maltol | (Han et al. 2023) |
|  |  | 37-31E | < 10 µg/mL | | Methylthioadenosine | (Andreu-Pérez et al. 2010) |
|  |  | MeWo |  |  |  |  |
|  |  | SKMel 147 |  |  |  |  |
|  |  | SKMel 103 |  |  |  |  |
|  |  | UACC903 |  |  |  |  |
|  |  | Colo 829 |  |  |  |  |
| 7. | Gastric Cancer | BGC-823 | 5 µg/mL | | Norharman | (Zheng et al. 2006) |
|  |  | SGC-7901 | < 50 µg/mL | | β-Ionone | (Dong et al. 2013) |
| 8. | Lung Cancer | A549 | < 10 µg/mL | | 8-Hydroxyquinoline | (Balthazar et al. 2022) |
|  |  |  |  |  | β-Ionone | (Lee et al. 2013) |
| 9. | Brain Cancer | U-373 MG | 894 μg/mL | | Atropine | (Yahia et al. 2018) |
|  |  | T98G | > 100 µg/mL | | Kynurenic acid | (Walczak et al. 2014a) |
| 10. | Cervical Cancer | HeLa | > 10 µg/mL | | Norharman | (Zheng et al. 2006) |
| 11. | Kidney Cancer | Caki-2 | < 10 µg/mL | | Kynurenic acid | (Walczak et al. 2012) |

Table 2. Antioxidative compounds from *P. noxium*.

| Antioxidation Properties | | | | Compounds | | | References |
| --- | --- | --- | --- | --- | --- | --- | --- |
| No. | Antioxidant assay | | IC_50_ |  |  |  |  |
| 1. | DPPH Radical-Scavenging Activity | | < 50 µg/mL | | 8-Gingerol | | (Dugasani et al. 2010) |
|  |  |  |  |  | trans-Clovamide | | (Kolodziejczyk-Czepas et al. 2017) |
|  |  |  | > 50 µg/mL | | (-)-Caryophyllene oxide | | (Karakaya et al. 2020) |
|  |  |  |  |  | Betaine | | (Zhang et al. 2016) |
|  |  |  |  |  | Loliolide | | (Yang et al. 2011) |
| 2. | OH Radical Scavenging Assay | | < 50 µg/mL | | Kynurenic acid | | (Lugo-Huitrón et al. 2011) |
|  |  |  | > 50 µg/mL | | Betaine | | (Zhang et al. 2016) |
|  |  |  | N/A | | L-Ergothioneine | | (Franzoni et al. 2006) |
| 3. | ONOO^-^ Scavenging Assay | | < 50 µg/mL | | trans-Clovamide | | (Kolodziejczyk-Czepas et al. 2017) |
|  |  |  | > 50 µg/mL | | Kynurenic acid | | (Lugo-Huitrón et al. 2011) |
|  |  |  | N/A | | L-Ergothioneine | | (Franzoni et al. 2006) |
| 4. | O^2-^ Radical Scavenging Assay | | < 50 µg/mL | | Kynurenic acid | | (Lugo-Huitrón et al. 2011) |
|  |  |  | > 50 µg/mL | | Betaine | | (Zhang et al. 2016) |
| 5. | Trolox Equivalent Antioxidant Capacity (TEAC) Assay | | < 50 µg/mL | | Carbidopa | | (Colamartino et al. 2015) |
|  |  |  | > 50 µg/mL | | Betaine | | (Zhang et al. 2016) |
| 6. | Ferric Reducing Antioxidant Potential Assay | | > 100 µg/mL | | Betaine | | (Zhang et al. 2016) |
| 7. | Hydrogen Peroxide Scavenging Assay | | < 50 µg/mL | | Loliolide | | (Yang et al. 2011) |
| 8. | Peroxyl Radical Scavenging Assay | | N/A | | L-Ergothioneine | | (Franzoni et al. 2006) |
|  | Antioxidant Activity Assay | Mechanism | | | |  |  |
| 9. | MDA Levels | Decreased in MDA levels | | | | Acetyl-L-carnitine | (Liu et al. 2004) |
|  |  |  |  |  |  | Isoleucine | (Zhao et al. 2014) |
|  |  |  |  |  |  | Ligustrazine | (Liu et al. 2008) |
|  |  |  |  |  |  | Maltol | (Wang et al. 2019) |
|  |  |  |  |  |  | Nicotinic acid | (Kaplon et al. 2014) |
|  |  |  |  |  |  | Trigonelline | (Khalili et al. 2018) |
| 10. | SOD Levels | Increased in SOD | | | | Carbidopa | (Colamartino et al. 2015) |
|  |  |  |  |  |  | Isoleucine | (Zhao et al. 2014) |
|  |  |  |  |  |  | Ligustrazine | (Liu et al. 2008) |
|  |  |  |  |  |  | Maltol | (Wang et al. 2019) |
|  |  |  |  |  |  | Trigonelline | (Khalili et al. 2018) |
| 11. | Lipid/Protein Peroxidation Assay | Decreased in Lipid/Protein Peroxidation Levels | | | | Kynurenic acid | (Lugo-Huitrón et al. 2011) |
|  |  |  |  |  |  | Methylthioadenosine | (Simile et al. 2001) |
|  |  |  |  |  |  | Nicotinamide | (Kamat & Devasagayam 1999) |
|  |  |  |  |  |  | Nicotinic acid | (Kaplon et al. 2014) |
|  |  |  |  |  |  | Thiamine | (Lukienko et al. 2000) |
| 12. | ROS Assay | Decreased in ROS | | | | Sedanolide | (Tabei et al. 2023) |
| 13. | Nrf2 Transcription Factor Assay | Upregulation of Nrf2 | | | | 5-Methoxyindoleacetic acid | (Saeedi et al. 2020) |
|  |  |  |  |  |  | Isoleucine | (Zhao et al. 2014) |
|  |  |  |  |  |  | Sedanolide | (Tabei et al. 2023) |
| 14. | Nitric Oxide Synthase Activity Assay | Activation of endothelial nitric oxide synthase | | | | Adenosine | (Xu et al. 2005) |

Table 3. Anti-inflammatory compounds identified from *P. noxium*.

| No. | Anti-inflammatory Assay | | Mechanism | Compounds | References |
| --- | --- | --- | --- | --- | --- |
|  | Cells stimulation for anti-inflammatory studies | | Downregulation of Pro-inflammatory Makers/Mediators/Cytokine |  |  |
|  | Cells | Activator |  |  |  |
| 1. | RAW264.7 macrophages | Lipopolysaccharide | NF-κB/IL-1β/TNF-α/IL-6/PGE2/COX-2 | Loliolide | (Jayawardena et al. 2021) |
|  |  |  | NF-κB/TNF-α | Methylthioadenosine | (Hevia et al. 2004) |
|  |  |  | NF-κB/TNF-α/IL-1 β /IL-6/MCP-1 | N-(3-oxodecanoyl)-L-homoserine lactone | (Qin et al. 2020) |
|  |  |  | TNF-α/IL-1β | Neosaxitoxin | (Montero et al. 2020) |
|  |  |  | IL-2/IL-8 | Solanidine | (Kenny et al. 2013) |
|  |  |  | TNF-α/IL-6/VEGF | Nicotinamide | (Yanez et al. 2019) |
| 2. | BV2 murine microglial cells |  | NF-κB/TNF-α/IL-1β/IL-6 | 8-Gingerol | (Ho et al. 2013) |
| 3. | Bone marrow-derived macrophages |  | TNF | 3-Indoleacrylic acid | (Wlodarska et al. 2017) |
| 4. | Human Umbilical Vein Endothelial Cells |  | TNF-α/MCP-1 | Kynurenic acid | (Lee et al. 2019) |
| 5. | Primary peripheral blood mononuclear cells & U-937 macrophages |  | NF-κB/TNF-α/IL-6 | Nonivamide | (Walker et al. 2017) |
| 6. | Male Wistar rats |  | NF-κB/TNF-α/IL-1β | Parthenolide | (Rummel et al. 2011) |
| 7. | Male C57BL/6J mice |  | TNF-α | Atropine | (Fuentes et al. 2008) |
| 8. | Male albino Wistar rats |  | NF-κB/TLR4/TNF-α | Trigonelline | (Khalili et al. 2018) |
| 9. | Human keratinocyte cell line | TNF-α | TNF-α/IL-6/IL-1β/IL-8 | (+)-ar-Tumerone | (Yang et al. 2020a) |
| 10. | Human Umbilical Vein Endothelial Cells | Uremic serum | MCP-1/VEGF | 4-Phenylbutyric acid | (Zeng et al. 2014) |
| 11. | 3T3-L1 cells | TNF-α | TNF-α/IL-6 | L-Ergothioneine | (Ito et al. 2011) |
| 12. | Human articular cartilage tissue with osteoarthritis | - | NF-κB/IL-1β/TNF-α/IL-6 | Maltol | (Lu et al. 2021) |
| 13. | Normal human aortic endothelial cells | TNF-α | NF-κB/TNF-α/MCP-1 | Nicotinic acid | (Ganji et al. 2009) |
| 14. | Human monocytes | Phorbol 12-myristate 13-acetate | NF-κB/TNF-α/IL-6 | trans-Clovamide | (Zeng et al. 2011) |
| 15. | Male Wistar Rats | Arsenic | NF-κB/IL-1β/IL-6 | Acetyl-L-carnitine | (Bodaghi-Namileh et al. 2018) |
| 16. | Male Sprague-Dawley Rats | Monocrotaline | MCP-1/ET-1/NF-κB/IL-1β/TNF-α | Betaine | (Yang et al. 2018) |
| 17. | Obese Ldlr-/-.Leiden mice | - | IL-1β/ TNF-α/CCR2/CXCL12 | Isoleucine | (Gart et al. 2022) |
| 18. | Male C57BL6/J mice | Controlled cortical impact induced Traumatic Brain Injury | IL-1β/IFN-B1/Nox2 | N-acetyl-L-leucine | (Hegdekar et al. 2021) |
|  | Cells stimulation for inflammatory studies | | Upregulation of Anti-inflammatory Makers/Mediators/Cytokine |  |  |
|  | Cells | Activator |  |  |  |
| 19. | RAW264.7 macrophages | Lipopolysaccharide | IL-10 | Methylthioadenosine | (Hevia et al. 2004) |
| 20. | Human monocytic cell line (THP-1) |  | MRC-1/IL-10 | Nicotinamide | (Yanez et al. 2019) |
| 21. | Bone marrow-derived macrophages |  | IL-10 | 3-Indoleacrylic acid | (Wlodarska et al. 2017) |
| 22. | Male C57BL/6J |  | IL-10 | Atropine | (Fuentes et al. 2008) |
| 23. | Male C57BL6/J mice | Controlled cortical impact (CCI) induced Traumatic Brain Injury | Socs3/YM-1/IL4ra/Arg-1 | N-Acetyl-L-leucine | (Hegdekar et al. 2021) |
| 24. | Obese Ldlr-/-.Leiden mice | - | IL-4 | Isoleucine | (Gart et al. 2022) |
| 25. | Nitric Oxide (NO) Assay | | Decreased in NO production | Loliolide | (Jayawardena et al. 2021) |
|  |  |  |  | Methylthioadenosine | (Hevia et al. 2004) |
|  |  |  |  | Neosaxitoxin | (Montero et al. 2020) |
|  |  |  |  | Solanidine | (Kenny et al. 2013) |
| 26. | Carrageenan-Induced Paw Edema/Exudates | | Inhibition of edema | (-)-Caryophyllene oxide | (Chavan et al. 2010) |

Table 4. Antibacterial compounds identified from *P. noxium*.

| Antibacterial Properties | | | Compound Name | References |
| --- | --- | --- | --- | --- |
| No. | Bacteria | MIC |  |  |
| 1. | *Escherichia coli* | < 100 µg/mL | 3-Hydroxy-2-methylpyridine | (Cao et al. 2019) |
|  |  |  | 8-Hydroxyquinoline | (Narayana et al. 2008) |
|  |  |  | Crucigasterin E | (Ciavatta et al. 2010) |
|  |  |  | N-acetyltyramine | (Driche et al. 2022) |
|  |  |  | Trigonelline | (Özçelik et al. 2011) |
|  |  | N/A | Isoleucine | (Ren et al. 2019) |
|  |  |  | (+)-ar-Turmerone | (Lee 2006) |
| 2. | *Staphylococcus aureus* | < 100 µg/mL | 8-Hydroxyquinoline | (Narayana et al. 2008) |
|  |  |  | Norharman | (Zheng et al. 2005) |
|  |  |  | N-acetyltyramine | (Driche et al. 2022) |
|  |  |  | Trigonelline | (Özçelik et al. 2011) |
|  |  |  | 3-Hydroxy-2-methylpyridine | (Cao et al. 2019) |
|  |  | N/A | Acetanisole | (Bowles et al. 1995) |
| 3. | *Bacillus subtilis* | < 100 µg/mL | 8-Hydroxyquinoline | (Narayana et al. 2008) |
|  |  |  | Norharman | (Zheng et al. 2005) |
|  |  |  | N-acetyltyramine | (Driche et al. 2022) |
|  |  |  | Trigonelline | (Özçelik et al. 2011) |
| 4. | *Pseudomonas aeruginosa* | < 100 µg/mL | 8-Hydroxyquinoline | (Narayana et al. 2008) |
|  |  |  | N-acetyltyramine | (Driche et al. 2022) |
|  |  |  | Trigonelline | (Özçelik et al. 2011) |
| 5. | *Bacillus cereus* | < 50 µg/mL | 8-Hydroxyquinoline | (Narayana et al. 2008) |
| 6. | *Klebsiella pneumoniae* | < 50 µg/mL | 8-Hydroxyquinoline | (Narayana et al. 2008) |
|  |  |  | N-acetyltyramine | (Driche et al. 2022) |
|  |  |  | Trigonelline | (Özçelik et al. 2011) |
| 7. | *Ralstonia solanacearum* | N/A | Benzylideneacetone | (Ji et al. 2004) |
| 8. | *Agrobacterium tumefaciens* | < 100 µg/mL | Norharman | (Zheng et al. 2005) |
| 9. | *Enterococcus faecalis* | < 50 µg/mL | N-acetyltyramine | (Driche et al. 2022) |
|  |  |  | Trigonelline | (Özçelik et al. 2011) |
|  |  | > 50 µg/mL | 3-Hydroxy-2-methylpyridine | (Cao et al. 2019) |
| 10. | *Acinetobacter baumannii* | 8 µg/mL | Trigonelline | (Özçelik et al. 2011) |
| 11. | *Clostridium perfringens* | N/A | (+)-ar-Turmerone | (Lee 2006) |
| 12. | *Pectobacterium carotovorum* subsp. c*arotovorum* | N/A | Benzylideneacetone | (Ji et al. 2004) |
| 13. | *Salmonella enterica* | 256 µg/mL | 3-Hydroxy-2-methylpyridine | (Cao et al. 2019) |
| 14. | *Heliobacter pylori* | < 50 µg/mL | 4-Guanidinobutyric acid | (Hwang & Jeong 2012) |
| 15. | *Proteus vulgaris* | 25 µg/mL | 8-Hydroxyquinoline | (Narayana et al. 2008) |
| 16. | *Pseudomonas fluorescens* | 50 µg/mL |  |  |
| 17. | *Serratia marcescens* | 10 µg/mL |  |  |
| 18. | *Agrobacterium vitis* | N/A | Benzylideneacetone | (Ji et al. 2004) |
| 19. | *Pectobacterium carotovorum* subsp. *atrosepticum* |  |  |  |
| 20. | *Pseudomonas syringae* pv. *tabaci* |  |  |  |
| 21. | *Micrococcus luteus* | 100 µg/mL | Cyclo(L-Phenylalanyl-L-Prolyl) | (Qi et al. 2009) |
| 22. | *Loktanella hongkongensis* | 200 µg/mL |  |  |
| 23. | *Ruegeria* sp. |  |  |  |
| 24. | *Enterobacter cloacae* | 30 µg/mL | N-acetyltyramine | (Driche et al. 2022) |
| 25. | *Proteus mirabilis* | 8 µg/mL | Trigonelline | (Özçelik et al. 2011) |

Table 5. Antifungal compounds identified from *P. noxium*.

| Antifungal Properties | | | Compound Name | References |
| --- | --- | --- | --- | --- |
| No. | Fungal | MIC |  |  |
| 1. | *Candida albicans* | < 50 µg/mL | 8-Hydroxyquinoline | (Narayana et al. 2008) |
|  |  |  | Sedanolide | (Momin & Nair 2001) |
|  |  |  | Trigonelline | (Özçelik et al. 2011) |
|  |  | > 50 µg/mL | 3-Hydroxy-2-methylpyridine | (Cao et al. 2019) |
| 2. | *Aspergillus flavus* | < 50 µg/mL | (+)-ar-Tumerone | (Li et al. 2023) |
|  |  |  | 8-Hydroxyquinoline | (Narayana et al. 2008) |
|  |  | N/A | β-Ionone | (Wilson et al. 1981) |
| 3. | *Aspergillus niger* | < 100 µg/mL | 8-Hydroxyquinoline | (Narayana et al. 2008) |
| 4. | *Candida parapsilasis* | < 10 µg/mL | Trigonelline | (Özçelik et al. 2011) |
|  |  | > 10 µg/mL | Sedanolide | (Momin & Nair 2001) |
| 5. | *Fusarium oxysporum* | 10 µg/mL | 8-Hydroxyquinoline | (Narayana et al. 2008) |
|  |  | N/A | Carvone | (Morcia et al. 2012) |
| 6. | *Penicillium roqueforti* | > 3000 µg/mL | Cyclo(L-Phenylalanyl-L-Prolyl) | (Ström et al. 2002) |
| 7. | *Epidermophyton floccosum* | < 10 µg/mL | 8-Hydroxyquinoline | (Narayana et al. 2008) |
| 8. | *Alternaria brassicicola* | N/A | 6-Amyl-2-pyrone | (Fujita et al. 2021) |
| 9. | *Fusarium udum* | < 10 µg/mL | 8-Hydroxyquinoline | (Narayana et al. 2008) |
| 10. | *Penicillium citrinum* |  |  |  |
| 11. | *Alternaria alternata* | N/A | Carvone | (Morcia et al. 2012) |
| 12. | *Aspergillus tubingensis* |  |  |  |
| 13. | *Fusarium cerealis* |  |  |  |
| 14. | *Fusarium culmorum* |  |  |  |
| 15. | *Fusarium proliferatum* |  |  |  |
| 16. | *Fusarium sporotrichioides* |  |  |  |
| 17. | *Fusarium subglutinans* |  |  |  |
| 18. | *Fusarium verticillioides* |  |  |  |
| 19. | *Penicillium* sp. |  |  |  |
| 20. | *Microsporum gypseum* |  |  |  |
| 21. | *Penicillium avellaneum* |  |  |  |
| 22. | *Trichophyton mentagraphytes* | > 50 µg/mL |  |  |
| 23. | *Aspergillus fumigatus* | 20000 µg/mL | Cyclo(L-Phenylalanyl-L-Prolyl) | (Ström et al. 2002) |
| 24. | *Phytophthora megakarya* | > 70000 µg/mL | trans-Clovamide | (Knollenberg et al. 2020) |
| 25. | *Phytophthora palmivora* |  |  |  |
| 26. | *Aspergillus parasiticus* | N/A | β-Ionone | (Wilson et al. 1981) |

Table 6. Antiviral metabolites identified from *P. noxium*.

| Antiviral Properties | | | Compound Name | References |
| --- | --- | --- | --- | --- |
| No. | Virus | Concentration for 50% Inhibition |  |  |
| 1. | Herpes simplex virus type-1 (HSV-1) | < 10 µg/mL | Atropine | (Özçelik et al. 2011) |
|  |  |  | Trigonelline |  |
| 2. | Parainfluenza type-3 (PI-3) | < 10 µg/mL | Atropine | (Özçelik et al. 2011) |
|  |  |  | Trigonelline |  |
| 3. | Avian influenza virus A/Chicken/Egypt/M7217B/2013 (H5N1) | 3.27 µg/mL | trans-Clovamide | (El-Sharawy et al. 2017) |
| 4. | Dengue virus | < 10 µg/mL | Pancracine | (Masi et al. 2022) |
| 5. | Hepatitis A virus | 50 µg/mL | Atropine | (Biziagos et al. 1990) |
| 6. | Herpesvirus | N/A | Cytarabine | (Renis 1973) |
| 7. | Human immunodeficiency virus (HIV) | < 10 µg/mL | Pancracine | (Masi et al. 2022) |
| 8. | Rotavirus | > 1000 µg/mL | Isoleucine | (Mao et al. 2018) |

**References**

Ahmed EA, Alkuwayti MA, and Ibrahim H-IM. 2022. Atropine is a suppressor of Epithelial–Mesenchymal Transition (EMT) that reduces stemness in drug-resistant breast cancer cells. *International Journal of Molecular Sciences* 23:9849 DOI: 10.3390/ijms23179849

Andreu-Pérez P, Hernandez-Losa J, Moliné T, Gil R, Grueso J, Pujol A, Cortés J, Avila MA, and Recio JA. 2010. Methylthioadenosine (MTA) inhibits melanoma cell proliferation and in vivo tumor growth. *BMC Cancer* 10:1-11 DOI: 10.1186/1471-2407-10-265

Balthazar JD, Soosaimanickam MP, Emmanuel C, Krishnaraj T, Sheikh A, Alghafis SF, and Ibrahim H-IM. 2022. 8-Hydroxyquinoline a natural chelating agent from *Streptomyces* spp. inhibits A549 lung cancer cell lines via BCL2/STAT3 regulating pathways. *World Journal of Microbiology and Biotechnology* 38:182 DOI: 10.1007/s11274-022-03368-4

Bian Y, Yang L, Sheng W, Li Z, Xu Y, Li W, and Zeng L. 2021. Ligustrazine induces the colorectal cancer cells apoptosis via p53-dependent mitochondrial pathway and cell cycle arrest at the G0/G1 phase. *Annals of Palliative Medicine* 10:1578-1588 DOI: 10.21037/apm-20-288

Biziagos E, Crance J-M, Passagot J, and Deloince R. 1990. Inhibitory effects of atropine, protamine, and their combination on hepatitis A virus replication in PLC/PRF/5 cells. *Antimicrobial Agents and Chemotherapy* 34:1112-1117 DOI: 10.1128/aac.34.6.1112

Bodaghi-Namileh V, Sepand MR, Omidi A, Aghsami M, Seyednejad SA, Kasirzadeh S, and Sabzevari O. 2018. Acetyl-L-carnitine attenuates arsenic-induced liver injury by abrogation of mitochondrial dysfunction, inflammation, and apoptosis in rats. *Environmental Toxicology and Pharmacology* 58:11-20 DOI: 10.1016/j.etap.2017.12.005

Bowles B, Sackitey S, and Williams A. 1995. Inhibitory effects of flavor compounds on *Staphylococcus aureus* WRRC B124. *Journal of Food Safety* 15:337-347 DOI: 10.1111/j.1745-4565.1995.tb00144.x

Cao DT, Tran VH, Vu VN, Mai HDT, Le THM, Vu TQ, Nguyen HH, Chau VM, and Pham VC. 2019. Antimicrobial metabolites from a marine-derived Actinomycete *Streptomyces* sp. G278. *Natural Product Research* 33:3223-3230 DOI: 10.1080/14786419.2018.1468331

Chavan M, Wakte P, and Shinde D. 2010. Analgesic and anti-inflammatory activity of caryophyllene oxide from *Annona squamosa* L. bark. *Phytomedicine* 17:149-151 DOI: 10.1016/j.phymed.2009.05.016

Chen Z, Cai A, Zheng H, Huang H, Sun R, Cui X, Ye W, Yao Q, Chen R, and Kou L. 2020. Carbidopa suppresses prostate cancer via aryl hydrocarbon receptor-mediated ubiquitination and degradation of androgen receptor. *Oncogenesis* 9:49 DOI: 10.1038/s41389-020-0236-x

Chen Z, Xia X, Chen H, Huang H, An X, Sun M, Yao Q, Kim K, Zhang H, and Chu M. 2022. Carbidopa suppresses estrogen receptor-positive breast cancer via AhR-mediated proteasomal degradation of ERα. *Investigational New Drugs* 40:1216-1230 DOI: 10.1007/s10637-022-01289-5

Ciavatta ML, Manzo E, Nuzzo G, Villani G, Varcamonti M, and Gavagnin M. 2010. Crucigasterins A–E, antimicrobial amino alcohols from the Mediterranean colonial ascidian *Pseudodistoma crucigaster*. *Tetrahedron* 66:7533-7538 DOI: 10.1016/j.tet.2010.07.056

Colamartino M, Santoro M, Duranti G, Sabatini S, Ceci R, Testa A, Padua L, and Cozzi R. 2015. Evaluation of levodopa and carbidopa antioxidant activity in normal human lymphocytes in vitro: Implication for oxidative stress in Parkinson’s disease. *Neurotoxicity Research* 27:106-117 DOI: 10.1007/s12640-014-9495-7

Dong H-W, Wang K, Chang X-X, Jin F-F, Wang Q, Jiang X-F, Liu J-R, Wu Y-H, and Yang C. 2019. Beta-ionone-inhibited proliferation of breast cancer cells by inhibited COX-2 activity. *Archives of Toxicology* 93:2993-3003 DOI: 10.1007/s00204-019-02550-2

Dong H-W, Zhang S, Sun W-G, Liu Q, Ibla JC, Soriano SG, Han X-H, Liu L-X, Li M-S, and Liu J-R. 2013. β-Ionone arrests cell cycle of gastric carcinoma cancer cells by a MAPK pathway. *Archives of Toxicology* 87:1797-1808 DOI: 10.1007/s00204-013-1041-5

Driche EH, Badji B, Bijani C, Belghit S, Pont F, Mathieu F, and Zitouni A. 2022. A new saharan strain of *Streptomyces* sp. GSB-11 produces maculosin and N-acetyltyramine active against multidrug-resistant pathogenic bacteria. *Current Microbiology* 79:298 DOI: 10.1007/s00284-022-02994-3

Dugasani S, Pichika MR, Nadarajah VD, Balijepalli MK, Tandra S, and Korlakunta JN. 2010. Comparative antioxidant and anti-inflammatory effects of [6]-gingerol,[8]-gingerol,[10]-gingerol and [6]-shogaol. *Journal of Ethnopharmacology* 127:515-520 DOI: 10.1016/j.jep.2009.10.004

El-Sharawy RT, Elkhateeb A, Marzouk MM, Abd El-Latif RR, Abdelrazig SE, and El-Ansari MA. 2017. Antiviral and antiparasitic activities of clovamide: The major constituent of *Dichrostachys cinerea* (L.) Wight et Arn. *Journal of Applied Pharmaceutical Science* 7:219-223 DOI: 10.7324/JAPS.2017.70930

Faezizadeh Z, Gharib A, and Goudarzi M. 2016. Anti-proliferative and apoptotic effects of beta-ionone in human leukemia cell line K562. *Zahedan Journal of Research in Medical Scicences* 18 DOI: 10.17795/zjrms-7364

Franzoni F, Colognato R, Galetta F, Laurenza I, Barsotti M, Di Stefano R, Bocchetti R, Regoli F, Carpi A, and Balbarini A. 2006. An in vitro study on the free radical scavenging capacity of ergothioneine: Comparison with reduced glutathione, uric acid and trolox. *Biomedicine & Pharmacotherapy* 60:453-457 DOI: 10.1016/j.biopha.2006.07.015

Fuentes J, Fulton W, Nino D, Talamini M, and Maio AD. 2008. Atropine treatment modifies LPS-induced inflammatory response and increases survival. *Inflammation Research* 57:111-117 DOI: 10.1007/s00011-007-7134-y

Fujita R, Yokono M, Ube N, Okuda Y, Ushijima S, Fukushima-Sakuno E, Ueno K, Osaki-Oka K, and Ishihara A. 2021. Suppression of *Alternaria brassicicola* infection by volatile compounds from spent mushroom substrates. *Journal of Bioscience and Bioengineering* 132:25-32 DOI: 10.1016/j.jbiosc.2021.03.003

Ganji SH, Qin S, Zhang L, Kamanna VS, and Kashyap ML. 2009. Niacin inhibits vascular oxidative stress, redox-sensitive genes, and monocyte adhesion to human aortic endothelial cells. *Atherosclerosis* 202:68-75 DOI: 10.1016/j.atherosclerosis.2008.04.044

Gart E, van Duyvenvoorde W, Caspers MP, van Trigt N, Snabel J, Menke A, Keijer J, Salic K, Morrison MC, and Kleemann R. 2022. Intervention with isoleucine or valine corrects hyperinsulinemia and reduces intrahepatic diacylglycerols, liver steatosis, and inflammation in Ldlr−/−. Leiden mice with manifest obesity‐associated NASH. *The FASEB Journal* 36 DOI: 10.1096/fj.202200111R

Han N-R, Park H-J, Ko S-G, and Moon P-D. 2023. Maltol has anti-cancer effects via modulating PD-L1 signaling pathway in B16F10 cells. *Frontiers in Pharmacology* 14 DOI: 10.3389/fphar.2023.1255586

Hegdekar N, Lipinski MM, and Sarkar C. 2021. N-Acetyl-L-leucine improves functional recovery and attenuates cortical cell death and neuroinflammation after traumatic brain injury in mice. *Scientific Reports* 11:9249 DOI: 10.1038/s41598-021-88693-8

Hevia H, Varela-Rey M, Corrales FJ, Berasain C, Martínez-Chantar ML, Latasa UM, Lu SC, Mato JM, García-Trevijano ER, and Avila MA. 2004. 5′-Methylthioadenosine modulates the inflammatory response to endotoxin in mice and in rat hepatocytes. *Hepatology* 39:1088-1098 DOI: 10.1002/hep.20154

Ho S-C, Chang K-S, and Lin C-C. 2013. Anti-neuroinflammatory capacity of fresh ginger is attributed mainly to 10-gingerol. *Food Chemistry* 141:3183-3191 DOI: 10.1016/j.foodchem.2013.06.010

Hsieh S-L, Chen C-T, Wang J-J, Kuo Y-H, Li C-C, Hsieh L-C, and Wu C-C. 2015. Sedanolide induces autophagy through the PI3K, p53 and NF-κB signaling pathways in human liver cancer cells. *International Journal of Oncology* 47:2240-2246 DOI: 10.3892/ijo.2015.3206

Hu SM, Yao XH, Hao YH, Pan AH, and Zhou XW. 2020. 8‑Gingerol regulates colorectal cancer cell proliferation and migration through the EGFR/STAT/ERK pathway. *International Journal of Oncology* 56:390-397 DOI: 10.3892/ijo.2019.4934

Hwang IY, and Jeong CS. 2012. Inhibitory effects of 4-guanidinobutyric acid against gastric lesions. *Biomolecules & Therapeutics* 20:239 DOI: 10.4062/biomolther.2012.20.2.239

Ito T, Kato M, Tsuchida H, Harada E, Niwa T, and Osawa T. 2011. Ergothioneine as an anti-oxidative/anti-inflammatory component in several edible mushrooms. *Food Science and Technology Research* 17:103-110 DOI: 10.3136/fstr.17.103

Jayawardena TU, Kim H-S, Sanjeewa KA, Han EJ, Jee Y, Ahn G, Rho J-R, and Jeon Y-J. 2021. Loliolide, isolated from *Sargassum horneri*; bate LPS-induced inflammation via TLR mediated NF-κB, MAPK pathways in macrophages. *Algal Research* 56:102297 DOI: 10.1016/j.algal.2021.102297

Ji D, Yi Y, Kang G-H, Choi Y-H, Kim P, Baek N-I, and Kim Y. 2004. Identification of an antibacterial compound, benzylideneacetone, from *Xenorhabdus nematophila* against major plant-pathogenic bacteria. *FEMS Microbiology Letters* 239:241-248 DOI: 10.1016/j.femsle.2004.08.041

Jones S, Fernandes NV, Yeganehjoo H, Katuru R, Qu H, Yu Z, and Mo H. 2013. β-Ionone induces cell cycle arrest and apoptosis in human prostate tumor cells. *Nutrition and Cancer* 65:600-610 DOI: 10.1080/01635581.2013.776091

Kamat J, and Devasagayam T. 1999. Nicotinamide (vitamin B3) as an effective antioxidant against oxidative damage in rat brain mitochondria. *Redox Report* 4:179-184 DOI: 10.1179/135100099101534882

Kaplon RE, Gano LB, and Seals DR. 2014. Vascular endothelial function and oxidative stress are related to dietary niacin intake among healthy middle-aged and older adults. *Journal of Applied Physiology* 116:156-163 DOI: 10.1152/japplphysiol.00969.2013

Karakaya S, Yilmaz SV, Özdemir Ö, Koca M, Pınar NM, Demirci B, Yıldırım K, Sytar O, Turkez H, and Baser KHC. 2020. A caryophyllene oxide and other potential anticholinesterase and anticancer agent in *Salvia verticillata* subsp. *amasiaca* (Freyn & Bornm.) Bornm.(Lamiaceae). *Journal of Essential Oil Research* 32:512-525 DOI: 10.1080/10412905.2020.1813212

Kenny OM, McCarthy CM, Brunton NP, Hossain MB, Rai DK, Collins SG, Jones PW, Maguire AR, and O'Brien NM. 2013. Anti-inflammatory properties of potato glycoalkaloids in stimulated Jurkat and Raw 264.7 mouse macrophages. *Life Sciences* 92:775-782 DOI: 10.1016/j.lfs.2013.02.006

Khalili M, Alavi M, Esmaeil-Jamaat E, Baluchnejadmojarad T, and Roghani M. 2018. Trigonelline mitigates lipopolysaccharide-induced learning and memory impairment in the rat due to its anti-oxidative and anti-inflammatory effect. *International Immunopharmacology* 61:355-362 DOI: 10.1016/j.intimp.2018.06.019

Kim SY, Moon KA, Jo HY, Jeong S, Seon SH, Jung E, Cho YS, Chun E, and Lee KY. 2012. Anti‐inflammatory effects of apocynin, an inhibitor of NADPH oxidase, in airway inflammation. *Immunology and Cell Biology* 90:441-448 DOI: 10.1038/icb.2011.60

Knollenberg BJ, Li G-X, Lambert JD, Maximova SN, and Guiltinan MJ. 2020. Clovamide, a hydroxycinnamic acid amide, is a resistance factor against *Phytophthora* spp. in *Theobroma cacao*. *Frontiers in Plant Science* 11:617520 DOI: 10.3389/fpls.2020.617520

Kolodziejczyk-Czepas J, Krzyżanowska-Kowalczyk J, Sieradzka M, Nowak P, and Stochmal A. 2017. Clovamide and clovamide-rich extracts of three *Trifolium* species as antioxidants and moderate antiplatelet agents in vitro. *Phytochemistry* 143:54-63 DOI: 10.1016/j.phytochem.2017.07.011

Lee H-S. 2006. Antimicrobial properties of turmeric (*Curcuma longa* L.) rhizome-derived ar-turmerone and curcumin. *Food Science and Biotechnology* 15:559-563

Lee SM, Kim YS, Jang WJ, Rakib AM, Oh TW, Kim BH, Kim SY, Kim JO, and Ha YL. 2013. Anti-proliferative effects of β-ionone on human lung cancer A-549 cells. *Journal of Life Science* 23:1351-1359 DOI: 10.5352/JLS.2013.23.11.1351

Lee T, Park HS, Jeong JH, and Jung TW. 2019. Kynurenic acid attenuates pro-inflammatory reactions in lipopolysaccharide-stimulated endothelial cells through the PPARδ/HO-1-dependent pathway. *Molecular and Cellular Endocrinology* 495:110510 DOI: 10.1016/j.mce.2019.110510

Li W, Su X-m, Han Y, Xu Q, Zhang J, Wang Z, and Wang Y-p. 2015. Maltol, a Maillard reaction product, exerts anti-tumor efficacy in H22 tumor-bearing mice via improving immune function and inducing apoptosis. *RSC Advances* 5:101850-101859 DOI: 10.1039/C5RA17960B

Li Z, Sun Y, Gu L, Wang Y, Xu M, Zhou Y, Hu Y, and Ma W. 2023. Ar-turmerone suppresses *Aspergillus flavus* growth and aflatoxin accumulation: Finding a new antifungal agent based on stored maize. *Food Research International* 168:112735 DOI: 10.1016/j.foodres.2023.112735

Liu J, Head E, Kuratsune H, Cotman CW, and Ames BN. 2004. Comparison of the effects of L‐carnitine and acetyl‐L‐carnitine on carnitine levels, ambulatory activity, and oxidative stress biomarkers in the brain of old rats. *Annals of the New York Academy of Sciences* 1033:117-131 DOI: 10.1196/annals.1320.011

Liu M, Li W, Ma H, Yang X, Liu A, and Ji C. 2022. Formulation of a novel anti-leukemia drug and evaluation of its therapeutic effects in comparison with cytarabine. *Arabian Journal of Chemistry* 15:103690 DOI: 10.1016/j.arabjc.2022.103690

Liu X-h, Li J, Li Q-x, Ai Y-x, and Zhang L. 2008. Protective effects of ligustrazine on cisplatin-induced oxidative stress, apoptosis and nephrotoxicity in rats. *Environmental Toxicology and Pharmacology* 26:49-55 DOI: 10.1016/j.etap.2008.01.006

Lu H, Fu C, Kong S, Wang X, Sun L, Lin Z, Luo P, and Jin H. 2021. Maltol prevents the progression of osteoarthritis by targeting PI3K/Akt/NF‐κB pathway: In vitro and in vivo studies. *Journal of Cellular and Molecular Medicine* 25:499-509 DOI: 10.1111/jcmm.16104

Lugo-Huitrón R, Blanco-Ayala T, Ugalde-Muñiz P, Carrillo-Mora P, Pedraza-Chaverrí J, Silva-Adaya D, Maldonado P, Torres I, Pinzón E, and Ortiz-Islas E. 2011. On the antioxidant properties of kynurenic acid: Free radical scavenging activity and inhibition of oxidative stress. *Neurotoxicology and Teratology* 33:538-547 DOI: 10.1016/j.ntt.2011.07.002

Lukienko P, Mel'Nichenko N, Zverinskii I, and Zabrodskaya S. 2000. Antioxidant properties of thiamine. *Bulletin of Experimental Biology and Medicine* 130:874-876 DOI: 10.1007/BF02682257

Mao X, Gu C, Ren M, Chen D, Yu B, He J, Yu J, Zheng P, Luo J, and Luo Y. 2018. L-isoleucine administration alleviates rotavirus infection and immune response in the weaned piglet model. *Frontiers in Immunology* 9:1654 DOI: 10.3389/fimmu.2018.01654

Masi M, Di Lecce R, Mérindol N, Girard M-P, Berthoux L, Desgagné-Penix I, Calabrò V, and Evidente A. 2022. Cytotoxicity and antiviral properties of alkaloids isolated from *Pancratium maritimum*. *Toxins* 14:262 DOI: 10.3390/toxins14040262

Momin RA, and Nair MG. 2001. Mosquitocidal, nematicidal, and antifungal compounds from *Apium graveolens* L. seeds. *Journal of Agricultural and Food Chemistry* 49:142-145 DOI: 10.1021/jf001052a

Montero MC, Del Campo M, Bono M, Simon MV, Guerrero J, and Lagos N. 2020. Neosaxitoxin inhibits the expression of inflammation markers of the M1 phenotype in macrophages. *Marine Drugs* 18:283 DOI: 10.3390/md18060283

Morcia C, Malnati M, and Terzi V. 2012. In vitro antifungal activity of terpinen-4-ol, eugenol, carvone, 1, 8-cineole (eucalyptol) and thymol against mycotoxigenic plant pathogens. *Food Additives & Contaminants: Part A* 29:415-422 DOI: 10.1080/19440049.2011.643458

Murata K, and Moriyama M. 2007. Isoleucine, an essential amino acid, prevents liver metastases of colon cancer by antiangiogenesis. *Cancer Research* 67:3263-3268 DOI: 10.1158/0008-5472.CAN-06-3739

Narayana KJ, Prabhakar P, Vijayalakshmi M, Venkateswarlu Y, and Krishna PS. 2008. Study on bioactive compounds from *Streptomyces* sp. ANU 6277. *Polish Journal of Microbiology* 57:35

Özçelik B, Kartal M, and Orhan I. 2011. Cytotoxicity, antiviral and antimicrobial activities of alkaloids, flavonoids, and phenolic acids. *Pharmaceutical Biology* 49:396-402 DOI: 10.3109/13880209.2010.519390

Pan J, Shang J-F, Jiang G-Q, and Yang Z-X. 2015. Ligustrazine induces apoptosis of breast cancer cells in vitro and in vivo. *Journal of Cancer Research and Therapeutics* 11:454-458 DOI: 10.4103/0973-1482.147378

Qi S-H, Xu Y, Xiong H-R, Qian P-Y, and Zhang S. 2009. Antifouling and antibacterial compounds from a marine fungus *Cladosporium* sp. F14. *World Journal of Microbiology and Biotechnology* 25:399-406 DOI: 10.1007/s11274-008-9904-2

Qin K, Liu J, Fu K, Han S, Chen X, and Zhou L. 2020. Quorum sensing molecule N-3-oxodecanoyl-L-homoserine lactone (3-oxo-C 10-HSL) inhibits lipopolysaccharide-induced inflammatory responses of RAW264. 7 macrophages. *Chinese Journal of Cellular and Molecular Immunology* 36:776-781

Ren M, Cai S, Zhou T, Zhang S, Li S, Jin E, Che C, Zeng X, Zhang T, and Qiao S. 2019. Isoleucine attenuates infection induced by *E. coli* challenge through the modulation of intestinal endogenous antimicrobial peptide expression and the inhibition of the increase in plasma endotoxin and IL-6 in weaned pigs. *Food & Function* 10:3535-3542 DOI: 10.1039/C9FO00218A

Renis HE. 1973. Antiviral activity of cytarabine in herpesvirus–infected rats. *Antimicrobial Agents and Chemotherapy* 4:439-444 DOI: 10.1128/aac.4.4.439

Rummel C, Gerstberger R, Roth J, and Hübschle T. 2011. Parthenolide attenuates LPS-induced fever, circulating cytokines and markers of brain inflammation in rats. *Cytokine* 56:739-748 DOI: 10.1016/j.cyto.2011.09.022

Saeedi BJ, Liu KH, Owens JA, Hunter-Chang S, Camacho MC, Eboka RU, Chandrasekharan B, Baker NF, Darby TM, and Robinson BS. 2020. Gut-resident *Lactobacilli* activate hepatic Nrf2 and protect against oxidative liver injury. *Cell Metabolism* 31:956-968. e955 DOI: 10.1016/j.cmet.2020.03.006

Simile MM, Banni S, Angioni E, Carta G, De Miglio MR, Muroni MR, Calvisi DF, Carru A, Pascale RM, and Feo F. 2001. 5′-Methylthioadenosine administration prevents lipid peroxidation and fibrogenesis induced in rat liver by carbon-tetrachloride intoxication. *Journal of Hepatology* 34:386-394 DOI: 10.1016/S0168-8278(00)00078-7

Ström K, Sjögren Jr, Broberg A, and Schnürer J. 2002. *Lactobacillus plantarum* MiLAB 393 produces the antifungal cyclic dipeptides cyclo (L-Phe-L-Pro) and cyclo (L-Phe-trans-4-OH-L-Pro) and 3-phenyllactic acid. *Applied and Environmental Microbiology* 68:4322-4327 DOI: 10.1128/AEM.68.9.4322-4327.2002

Sultan N, Othman AI, El-Missiry MA, Mohamed AF, and Shabana SM. 2019. Assessment of the anticancer activity of caryophyllene oxide against breast cancer cell line and related genetic alterations: In vitro study. *Journal of Environmental Sciences Mansoura University* 48:87-94 DOI: 10.21608/joese.2019.158392

Tabei Y, Abe H, Suzuki S, Takeda N, Arai J-i, and Nakajima Y. 2023. Sedanolide activates KEAP1–NRF2 pathway and ameliorates hydrogen peroxide-induced apoptotic cell death. *International Journal of Molecular Sciences* 24:16532 DOI: 10.3390/ijms242216532

Walczak K, Deneka-Hannemann S, Jarosz B, Zgrajka W, Stoma F, Trojanowski T, Turski WA, and Rzeski W. 2014a. Kynurenic acid inhibits proliferation and migration of human glioblastoma T98G cells. *Pharmacological Reports* 66:130-136 DOI: 10.1016/j.pharep.2013.06.007

Walczak K, Turski WA, and Rajtar G. 2014b. Kynurenic acid inhibits colon cancer proliferation in vitro: Effects on signaling pathways. *Amino Acids* 46:2393-2401 DOI: 10.1007/s00726-014-1790-3

Walczak K, Żurawska M, Kiś J, Starownik R, Zgrajka W, Bar K, Turski WA, and Rzeski W. 2012. Kynurenic acid in human renal cell carcinoma: its antiproliferative and antimigrative action on Caki-2 cells. *Amino Acids* 43:1663-1670 DOI: 10.1007/s00726-012-1247-5

Walker J, Ley JP, Schwerzler J, Lieder B, Beltran L, Ziemba PM, Hatt H, Hans J, Widder S, and Krammer GE. 2017. Nonivamide, a capsaicin analogue, exhibits anti‐inflammatory properties in peripheral blood mononuclear cells and U‐937 macrophages. *Molecular Nutrition & Food Research* 61:1600474 DOI: 10.1002/mnfr.201600474

Wang Z, Hao W, Hu J, Mi X, Han Y, Ren S, Jiang S, Wang Y, Li X, and Li W. 2019. Maltol improves APAP-induced hepatotoxicity by inhibiting oxidative stress and inflammation response via NF-κB and PI3K/Akt signal pathways. *Antioxidants* 8:395 DOI: 10.3390/antiox8090395

Wen J, You K-R, Lee S-Y, Song C-H, and Kim D-G. 2002. Oxidative stress-mediated apoptosis: the anticancer effect of the sesquiterpene lactone parthenolide. *Journal of Biological Chemistry* 277:38954-38964 DOI: 10.1074/jbc.M203842200

Wilson D, Gueldner R, McKinney J, Lievsay R, Evans B, and Hill R. 1981. Effect of β-ionone on *Aspergillus flavus* and *Aspergillus parasiticus* growth, sporulation, morphology and aflatoxin production. *Journal of the American Oil Chemists’ Society* 58:A959-A961 DOI: 10.1007/BF02679300

Wlodarska M, Luo C, Kolde R, d’Hennezel E, Annand JW, Heim CE, Krastel P, Schmitt EK, Omar AS, and Creasey EA. 2017. Indoleacrylic acid produced by commensal *Peptostreptococcus* species suppresses inflammation. *Cell Host & Microbe* 22:25-37. e26 DOI: 10.1016/j.chom.2017.06.007

Xu Y, Ryu S, Lee Y-K, and Lee H-J. 2020. Brassicasterol from edible aquacultural *Hippocampus abdominalis* exerts an anti-cancer effect by dual-targeting AKT and AR signaling in prostate cancer. *Biomedicines* 8:370 DOI: 10.3390/biomedicines8090370

Xu Z, Park S-S, Mueller RA, Bagnell RC, Patterson C, and Boysen PG. 2005. Adenosine produces nitric oxide and prevents mitochondrial oxidant damage in rat cardiomyocytes. *Cardiovascular Research* 65:803-812 DOI: 10.1016/j.cardiores.2004.12.004

Yahia M, Benhouda A, and Haba H. 2018. New biological anticancer activities of atropine isolated from Algerian *Hyoscyamus albus*’s leaves. *Pharmacologyonline* 3:286-296

Yanez M, Jhanji M, Murphy K, Gower RM, Sajish M, and Jabbarzadeh E. 2019. Nicotinamide augments the anti-inflammatory properties of resveratrol through PARP1 activation. *Scientific Reports* 9:10219 DOI: 10.1038/s41598-019-46678-8

Yang J-m, Zhou R, Zhang M, Tan H-r, and Yu J-q. 2018. Betaine attenuates monocrotaline-induced pulmonary arterial hypertension in rats via inhibiting inflammatory response. *Molecules* 23:1274 DOI: 10.3390/molecules23061274

Yang MH, Ha IJ, Ahn J, Kim C-K, Lee M, and Ahn KS. 2023. Potential function of loliolide as a novel blocker of epithelial-mesenchymal transition in colorectal and breast cancer cells. *Cellular Signalling* 105:110610 DOI: 10.1016/j.cellsig.2023.110610

Yang S, Liu J, Jiao J, and Jiao L. 2020a. Ar-turmerone exerts anti-proliferative and anti-inflammatory activities in HaCaT keratinocytes by inactivating hedgehog pathway. *Inflammation* 43:478-486 DOI: 10.1007/s10753-019-01131-w

Yang W, Liu S, Li Y, Wang Y, Deng Y, Sun W, Huang H, Xie J, He A, and Chen H. 2020b. Pyridoxine induces monocyte-macrophages death as specific treatment of acute myeloid leukemia. *Cancer Letters* 492:96-105 DOI: 10.1016/j.canlet.2020.08.018

Yang X, Kang M-C, Lee K-W, Kang S-M, Lee W-W, and Jeon Y-J. 2011. Antioxidant activity and cell protective effect of loliolide isolated from *Sargassum ringgoldianum* subsp. *coreanum*. *Algae* 26:201-208 DOI: 10.4490/algae.2011.26.2.201

Zeng H, Locatelli M, Bardelli C, Amoruso A, Coisson JD, Travaglia F, Arlorio M, and Brunelleschi S. 2011. Anti-inflammatory properties of clovamide and *Theobroma cacao* phenolic extracts in human monocytes: Evaluation of respiratory burst, cytokine release, NF-κB activation, and PPARγ modulation. *Journal of Agricultural and Food chemistry* 59:5342-5350 DOI: 10.1021/jf2005386

Zeng W, Guo Y-H, Qi W, Chen J-G, Yang L-L, Luo Z-F, Mu J, and Feng B. 2014. 4-Phenylbutyric acid suppresses inflammation through regulation of endoplasmic reticulum stress of endothelial cells stimulated by uremic serum. *Life Sciences* 103:15-24 DOI: 10.1016/j.lfs.2014.03.007

Zhang M, Zhang H, Li H, Lai F, Li X, Tang Y, Min T, and Wu H. 2016. Antioxidant mechanism of betaine without free radical scavenging ability. *Journal of Agricultural and Food Chemistry* 64:7921-7930 DOI: 10.1021/acs.jafc.6b03592

Zhao J, Feng L, Liu Y, Jiang W, Wu P, Jiang J, Zhang Y, and Zhou X. 2014. Effect of dietary isoleucine on the immunity, antioxidant status, tight junctions and microflora in the intestine of juvenile Jian carp (C*yprinus carpio* var. Jian). *Fish & Shellfish Immunology* 41:663-673 DOI: 10.1016/j.fsi.2014.10.002

Zheng L, Chen H, Han X, Lin W, and Yan X. 2005. Antimicrobial screening and active compound isolation from marine bacterium NJ6-3-1 associated with the sponge *Hymeniacidon perleve*. *World Journal of Microbiology and Biotechnology* 21:201-206 DOI: 10.1007/s11274-004-3318-6

Zheng L, Yan X, Han X, Chen H, Lin W, Lee FS, and Wang X. 2006. Identification of norharman as the cytotoxic compound produced by the sponge (*Hymeniacidon perleve*)‐associated marine bacterium *Pseudoalteromonas piscicida* and its apoptotic effect on cancer cells. *Biotechnology and Applied Biochemistry* 44:135-142 DOI: 10.1042/BA20050176
