# Supplementary material for: A double agent? Unveiling the chemical profile of the pathogenic fungus Pyrrhoderma noxium as an endophyte in true mangroves
Source: PeerJ. 2026 Feb 20;14:e20826. doi: 10.7717/peerj.20826 (PMC12927600; doi:10.7717/peerj.20826)
Supplement: Supplemental Information 5 — The total ion chromatograms acquired from LC-MS/MS [file peerj-14-20826-s005.docx]

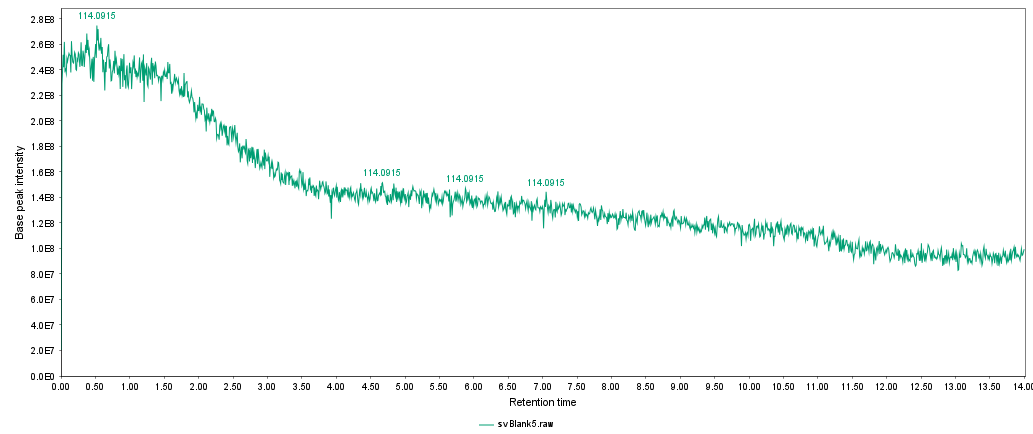

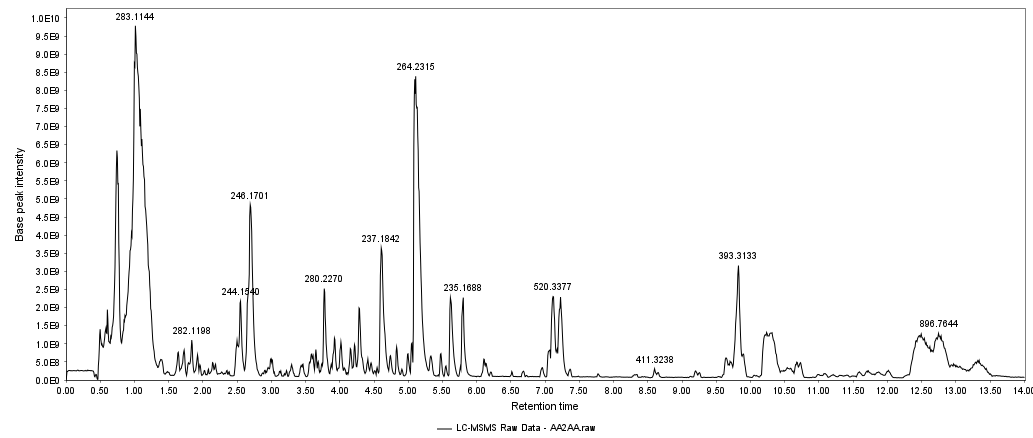

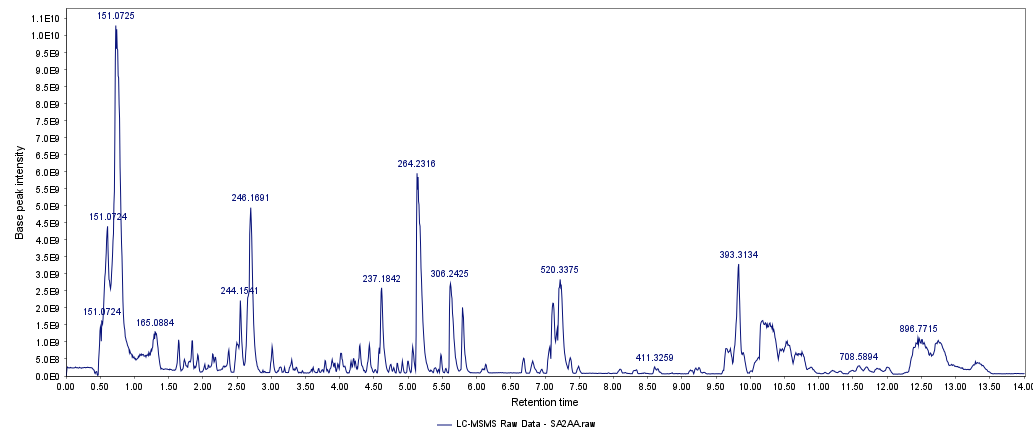

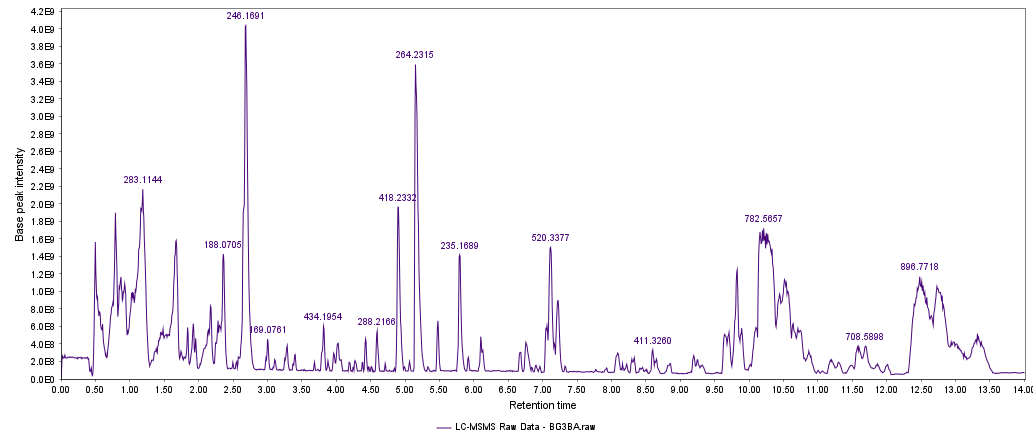


*AA2AA*

*SA2AA*

*BG3BA*

Solvent Blank

Supplementary E: Total ion chromatograms acquired from the LC-MS/MS analysis of solvent blank, *AA2AA*, *SA2AA*, and *BG3BA*
